# Exploring the predictive factors of heart disease using rare association rule mining

**DOI:** 10.1038/s41598-024-69071-6

**Published:** 2024-08-06

**Authors:** Sadeq Darrab, David Broneske, Gunter Saake

**Affiliations:** 1 Department of Computer Science, University of Magdeburg, 39106 Magdeburg, Germany; 2 German Centre for Higher Education Research and Science Studies, 30195 Hannover, Germany

**Keywords:** Heart disease, Cardiovascular risk factors, Early detection, Frequent association rules, Rare association rules, Interesting rules, Cardiology, Diseases

## Abstract

Cardiovascular diseases continue to be the leading cause of mortality worldwide, claiming a significant number of lives each year. Despite the advancements in predictive models, including logistic regression, neural networks, and random forests, these techniques often lack transparency and interpretability, limiting their practical application in clinical settings. To address this challenge, this research introduces EPFHD-RARMING, an innovative approach designed to enhance the understanding and predictability of heart disease through the discovery of rare and meaningful patterns. EPFHD-RARMING utilizes rare association rule mining to uncover hidden and unexpected rules that identify critical factors contributing to heart disease. This method is particularly adept at identifying high-risk patterns in individuals who appear healthy but may develop heart disease under certain conditions, thus facilitating early intervention and preventive measures. By integrating these insights with established feature engineering techniques, EPFHD-RARMING enhances its practical utility, enabling medical professionals to proactively manage patient care and tailor interventions to individual risk profiles. This study demonstrates the effectiveness of EPFHD-RARMING in providing a deeper, actionable understanding of the complex dynamics of heart disease. The model’s ability to identify and interpret rare patterns holds significant promise for advancing medical analytics and improving patient outcomes. Moreover, the applicability of EPFHD-RARMING extends beyond the healthcare domain, offering valuable insights in various fields where the discovery of rare patterns is critical, such as finance, marketing, and cybersecurity. This study conducts a comprehensive evaluation, which demonstrates the superior performance of EPFHD-RARMING compared to traditional predictive models in identifying key factors contributing to heart disease, in terms of interestingness, explainability, and comprehensiveness of insights. The results underscore the potential of this innovative approach to revolutionize our understanding and prediction of heart disease, ultimately contributing to more effective and personalized healthcare solutions. This research emphasizes the importance of rare association rule mining in medical analytics and paves the way for future studies to explore and utilize these techniques across diverse domains.

## Introduction

The World Health Organization (WHO) identifies cardiovascular diseases (CVDs) as the foremost cause of mortality globally, presenting a significant global health challenge^1^. This issue extends beyond high-income nations and is increasingly prevalent in low- and middle-income countries, where it imposes a substantial burden on individuals, health systems, and economies^2^. CVD risk factors are widespread throughout diverse populations, regardless of age, gender, socioeconomic status, or geographical location, highlighting the urgency of addressing this public health crisis.

While the WHO and other international organizations, such as the World Heart Federation (WHF), have been pivotal in raising awareness and shaping interventions, the global response to CVDs involves a complex interplay of surveillance, policy-making, and community engagement. Efforts to mitigate the impact of CVDs are multifaceted, ranging from the implementation of educational programs that promote healthy lifestyles to the strategic planning of global health initiatives^3^. The recognition of CVDs as a major global health issue by the WHO is supported by the widespread occurrence of CVDs and their risk factors, as well as the coordinated efforts of international health agencies to address this concern^3,4^. Addressing the challenge of CVDs requires a comprehensive approach that includes prevention, control, and global collaboration to reduce the associated morbidity and mortality^4^.

Machine learning (ML) techniques^5,6^ have shown immense potential across various domains, revolutionizing industries by providing enhanced data analysis, predictive capabilities, and automation. In finance, ML algorithms aid in fraud detection, risk assessment, and algorithmic trading. In marketing, AI-driven analytics offer personalized customer experiences and targeted advertising. The healthcare sector benefits from ML in areas such as disease prediction, patient monitoring, and personalized treatment plans.

In the context of heart disease, a significant global health concern leading to substantial morbidity and mortality rates, the importance of ML becomes particularly evident. The ability to process and analyze vast amounts of medical data enables the early detection of CVDs, allowing for timely and targeted interventions. These methods help in identifying subtle patterns and risk factors that may not be apparent through traditional diagnostic methods. For instance, ML algorithms can analyze electronic health records, imaging data, and genetic information to predict the likelihood of heart disease with remarkable accuracy. This predictive power not only aids in early diagnosis but also in the development of personalized treatment plans, ultimately improving patient outcomes and reducing healthcare costs. These techniques^7–10^ encompass a range of algorithms and models that can analyze complex medical data to identify patterns and predict health risks with high precision.

However, the interpretability and explainability of ML models^11^, particularly in the context of early detection and diagnosis of heart disease, remain significant concerns^12^. While ML models offer substantial advancements in identifying patterns within complex datasets for disease prediction, the opacity of these models can undermine trust and hinder their practical adoption in healthcare settings^12^. This lack of transparency may lead to ethical dilemmas and challenges in clinical decision-making, as healthcare practitioners and patients may not fully understand the reasoning behind AI-driven predictions.

Association rule mining (ARM) is a widely recognized and highly interpretable data mining technique that reveals hidden patterns and correlations among various factors^13^. Its prominence, ease of interpretation, and ability to extract valuable knowledge make it an excellent tool for real-world applications such as market basket analysis and web traffic analysis. However, despite its potential, ARM has not been widely adopted in the field of medicine. This is unfortunate, as association rules can identify every pattern in a given dataset, which is highly beneficial for clinical data analysis. By utilizing association rules, clinicians can quickly and automatically make well-informed diagnoses, extract valuable information, and develop essential knowledge bases.

Despite the advantages of ARM, it presents some challenges. One significant challenge is the generation of many irrelevant and repetitive rules. Furthermore, the most interesting rules often have low support values, referred to as rare rules. Low support thresholds can result in an overwhelming number of rules, complicating their management and analysis. Therefore, appropriate methods are necessary to determine the usefulness of the rules and to identify the most relevant ones^14^.

Rare association rule mining is crucial in enhancing the interpretability and explainability of data, particularly in the context of early detection and diagnosis of heart disease. By identifying infrequent but significant patterns within medical datasets, this technique can uncover subtle correlations between symptoms and heart disease that may not be apparent through traditional frequent pattern mining methods^15–17^. Interestingly, while rare association rule mining provides valuable insights, it also presents challenges such as the need for efficient algorithmic approaches to handle the balance between finding rare patterns and managing the vast number of potential rules generated^17,18^. Moreover, the application of this technique in medical datasets for heart disease diagnosis requires careful consideration of the rarity and relevance of the associations to ensure clinical utility. Hence, rare association rule mining plays a crucial role in the early detection and diagnosis of heart disease by enabling the discovery of rare but clinically relevant patterns that may improve interpretability and explainability. The technique’s ability to process and analyze complex medical data can lead to the identification of early indicators of heart disease, potentially leading to timely interventions and better patient outcomes. The challenges associated with this mining technique, such as creating a large amount of patterns, noise, and finding only the interesting rules, must be addressed to fully utilize its potential.

To address these challenges effectively, we propose EPFHD-RARMING (Exploring the Predictive Factors of Heart Disease using Rare Association Rule MinING). Our proposed solution aims to discover the factors contributing to heart disease without generating an excessive number of rules. Instead, we focus on generating only the relevant and interesting rules. The EPFHD-RARMING model incorporates two types of patterns, namely frequent and rare, to generate compelling and meaningful rules. Frequent patterns represent well-known or anticipated patterns, reflecting our existing knowledge (set of beliefs). By leveraging these patterns, we identify a set of rare rules that deviate from the established beliefs, with significantly lower support when additional features are considered. This approach allows us to explore predictive factors and their related symptoms leading to heart disease.

Feature selection has gained extensive attention in recent years due to its significant role in identifying the most important features for model predictions^19,20^. However, focusing solely on features and their impact on model predictions misses the importance of determining patterns associated with these features that lead to predictions. Therefore, in our proposed model, we emphasize not only feature selection but also the patterns that may indicate the development of heart disease when these features (symptoms, in our case study of heart disease) are present.

To the best of our knowledge, this study represents the first attempt to utilize simple yet powerful rule mining algorithms to extract symptoms and identify patterns indicative of future heart disease development. The rules generated by our model have the potential to significantly assist clinicians in making informed decisions for the early detection and treatment of heart disease. Our primary objective in this paper is to generate rules that are both insightful and applicable for predicting heart disease, thereby enhancing the explainability and transparency of predictive models.

The main motivations for our research are as follows:

- Early detection enhancement: Early detection of heart disease is crucial for reducing mortality rates. Current methods often rely on intricate models that lack interpretability. Our research aims to address this issue by employing rule mining algorithms that provide clear and actionable insights for clinicians.
- Improving clinical decision support: Clinicians require tools that facilitate rapid, well-informed decisions. Our model’s primary goal is to generate precise and relevant rules to support clinical decision-making processes, leading to improved patient outcomes.
- Enhancing data-driven approaches: With the rise of health data availability, there is an urgent need to effectively utilize this data. Our research focuses on uncovering patterns that are not immediately apparent through conventional analysis methods.
- Promoting transparency and trust in ML models: One of the significant challenges in ML models for healthcare is the lack of transparency. By using rule-based models, our research seeks to promote transparency, making it easier for healthcare professionals to understand and trust the outcomes.

For this paper, the main contributions are as follows:

- Innovative rule extraction: Our model, EPFHD-RARMING, specifically addresses the challenge of traditional association rule mining, which often produces an excessive number of low-support rules. It extracts a meaningful set of association rules from extensive data, focusing on those that are truly insightful and relevant, thus mitigating the common issue of rule quantity overwhelming quality.
- Critical factor identification: Our model effectively uncovers pivotal factors and symptoms of heart disease. Using advanced analytics, it prioritizes the most significant variables associated with cardiovascular risks, thereby enhancing early detection and intervention strategies.
- Predictive vulnerability analysis: This approach diverges from conventional models by identifying not only conditions directly linked to heart disease but also seemingly healthy states that may lead individuals to future health risks. This predictive analysis of vulnerabilities provides a deeper, more detailed understanding of potential health trajectories.
- Comprehensive data exploration through unsupervised learning: Utilizing the unsupervised tool of Association Rule Mining (ARM), our methodology offers a more complete exploration of datasets to identify overlooked patterns and factors. This comprehensive analysis aids in understanding the complex interactions between variables and heart disease. Furthermore, the rule-based approach enhances interpretability and usability, particularly in clinical settings, making the findings accessible and actionable for medical professionals.

This paper is organized as follows: “Background” defines association rules and related concepts. Section “Related work” reviews prior studies on predicting heart disease. Section “Dataset: heart disease” describes the characteristics of the heart disease dataset used in our research. Section “The proposed model: EPFHD-RARMING” details the proposed solution. Section "Experimental results" presents the results of our experiments with the medical dataset. Finally, Section "Discussion" concludes the paper with a discussion of future research directions.

## Background

To fully understand the proposed work, it is imperative to review the key concepts and definitions of association rule mining. Association rule mining^21^ is an unsupervised learning technique that seeks to uncover hidden patterns in a dataset. It is based on “if-then” logic, also known as association rules. A rule has two components: an antecedent (if) and a consequent (then), both of which are sets of items. For example, using a heart disease dataset, an association rule could be “if ’asymptomatic’, ’fasting blood sugar’ = 1, ’man’, then heart disease,” indicating that patients with chest pain type = ’asymptomatic’, fasting blood sugar = 1, and sex = ’man’ are more likely to have heart disease.

The process of association rule mining comprises two primary steps:

1. Identifying interesting patterns: A pattern is a set of items that appear together in a dataset and is considered interesting if it satisfies a threshold constraint.
2. Generating association rules: Association rules are generated from patterns obtained in the first step by splitting them into an antecedent and a consequent, and then evaluating their quality using metrics such as support, confidence, and lift.

To clarify concepts and terms associated with association rule mining, we provide the following formal definitions^22^.

Let $I = \{i_1, i_2, \ldots , i_n\}$ be a set of *n* unique items and $DB = \{ T_1, T_2, \ldots , T_m\}$ be a set of *m* transactions called a dataset. Each transaction $T_i \subseteq I$ consists of one or more items from *I*. An **association rule** is an implication of the form $X \rightarrow Y$, where $X \subseteq I$, $Y \subseteq I$, and $X \cap Y = \emptyset$. Here, *X* is called the **antecedent** of the rule and *Y* is called the **consequent** of the rule.

Two key metrics are widely used to assess the quality of an association rule: **support** and **confidence**.

1. **Support (Supp)**: This metric measures the frequency or proportion of transactions that contain both *X* and *Y*. It is defined as:
  1 $$\begin{aligned} \text {Supp}(X \rightarrow Y) = \frac{\sigma (X \cup Y)}{m} \end{aligned}$$
  where $\sigma (X \cup Y)$ is the number of transactions that contain both *X* and *Y*, and *m* is the number of transactions in the dataset. Additionally, the support of *X* is defined as:
  2 $$\begin{aligned} \text {Supp}(X) = \frac{\sigma (X)}{m} \end{aligned}$$
  where $\sigma (X)$ is the number of transactions that contain *X*.
2. **Confidence (Conf)**: This metric measures the conditional probability or strength of the rule, indicating how often *Y* appears in transactions that contain *X*. It is defined as:
  3 $$\begin{aligned} \text {Conf}(X \rightarrow Y) = \frac{\text {Supp}(X \rightarrow Y)}{\text {Supp}(X)} \end{aligned}$$
  where $\text {Supp}(X \rightarrow Y)$ is calculated as in Eq. (1), and $\text {Supp}(X)$ is calculated as in Eq. (2).

In general, a high support value indicates that the rule is broadly applicable across the dataset, while a high confidence value suggests that the rule is reliable and has strong predictive power.

Next, we review some key definitions related to association rule mining. These will help us better understand and interpret the results and implications of our proposed approach.

### Definition 1

(Frequent and rare pattern) A pattern *X* whose support satisfies a user-specified support threshold, *minSup*, is called a frequent pattern, such that $\text {Supp}(X) \ge minSup$. In contrast, a pattern *X* that does not satisfy *minSup* is called a rare pattern.

In our work, we utilize a formal definition of frequent patterns as collections of symptoms that frequently co-occur and are well-known and expected. These patterns may either signify the presence of heart disease or not, as both possibilities are reasonable given their frequent appearance in the dataset. On the other hand, rare patterns are characterized as sets of symptoms that generate unusual and infrequent rules. The most interesting patterns are those that are rare and may potentially contribute to the development of heart disease.

### Definition 2

(Strong association rule) An association rule $X \rightarrow Y$ is called strong if its $\text {Supp}$ and $\text {Conf}$ measures satisfy a specified minimum support threshold (*minSup*) and minimum confidence threshold (*minConf*), respectively.

### Definition 3

(Unexpected association rule^23^) An association rule (rare) $X \rightarrow Y$ is unexpected with respect to a known (frequent) rule $A \rightarrow B$ if the following conditions are met:

- The antecedents of the rules (i.e., *A* and *X*) are statistically significant on *DB* and have high similarity (above a given similarity threshold).
- The consequents of the rules (i.e., *B* and *Y*) are opposite to each other.

The traditional support-confidence model of frequent pattern generation has become widely popular because of its simplicity. Raw frequency counts and conditional probabilities are very helpful when it comes to supporting the claim and determining the confidence level. Despite this, the frequency of patterns does not always correspond to the most interesting patterns, as stated in a study^24^. Our approach addresses this limitation by using multiple commonly used metrics (lift, leverage, and conviction) to assess the generated rules. We combine these statistical measures with the previous definitions to find rules that are considered interesting. The metrics are defined as follows:

1. **Lift**^25^: It is a measure of how much more likely the antecedent and consequent of a rule are to occur together than expected if they were independent. It is defined as the ratio of the observed support of the rule to the expected support if the antecedent and consequent were independent. The formula for lift is:
  4 $$\begin{aligned} \text {lift}(X \rightarrow Y) = \frac{\text {Supp}(X \rightarrow Y)}{\text {Supp}(X) \cdot \text {Supp}(Y)} \end{aligned}$$
2. **Leverage (lev)**^25^: It is a measure of the difference between the observed support of the rule and the expected support if the antecedent and consequent were independent. It is defined as the difference between the observed support of the rule and the product of the supports of the antecedent and consequent. The formula for leverage is:
  5 $$\begin{aligned} \text {lev}(X \rightarrow Y) = \text {Supp}(X \rightarrow Y) - \text {Supp}(X) \cdot \text {Supp}(Y) \end{aligned}$$
3. **Conviction (conv)**^25^: It is a measure of how much the antecedent of a rule implies the consequent, or how often the rule would be incorrect if the antecedent and consequent were independent. It is defined as the ratio of the expected frequency of the antecedent occurring with an alternative consequent to the observed frequency of incorrect predictions. The formula for conviction is:
  6 $$\begin{aligned} \text {conv}(X \rightarrow Y) = \frac{1 - \text {Supp}(Y)}{1 - \text {Conf}(X \rightarrow Y)} \end{aligned}$$

**Figure 1:**
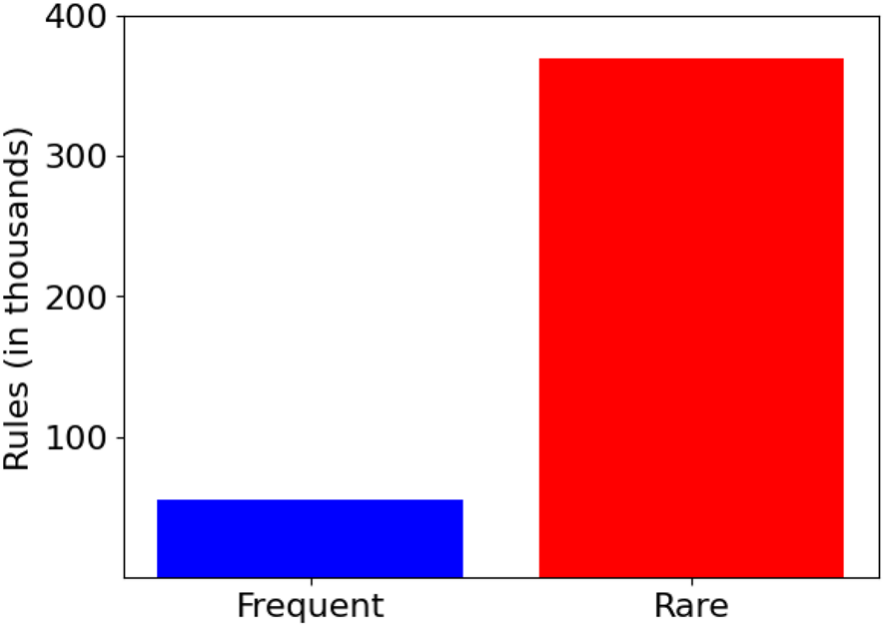
A comparison of the number of frequent and rare rules generated from the heart disease dataset.

Previous research has generally assumed that generating association rules from patterns is a straightforward process. However, this assumption is not necessarily accurate, as the primary objective of discovering patterns is to create meaningful rules. The sheer number of rules generated from patterns can be quite large, making analysis costly and impractical. This problem becomes even more complex when attempting to identify interesting rules among the rare ones. Therefore, identifying interesting rules from such a large set of rules is a significant challenge. For instance, our heart disease dataset consists of only 1190 transactions, yet it yields 448,981 rare and frequent rules, as illustrated in Fig. 1. Our proposed solution addresses this challenge by producing only the interesting and unexpected rules that can aid clinicians in determining the likelihood of heart disease based on the given symptoms.

Heart disease is one of the primary causes of death worldwide, underscoring the importance of early detection to save lives. However, accurately diagnosing and predicting the factors contributing to heart disease remains a significant challenge. This study aims to identify rules that comply with Definitions 1–3. Discovering such rules enables the extraction of surprising association rules from medical data. These surprising association rules are particularly valuable because they contradict established knowledge or expectations, thereby uncovering novel and insightful information. However, not all surprising rules are equally interesting or relevant. Therefore, a key question arises: how can we identify and evaluate the most compelling and significant rules for heart disease detection?

The objective of this paper is to propose a framework that addresses the question of generating and evaluating surprising association rules using a variety of metrics. The framework introduces various metrics, such as support, confidence, lift, leverage, and conviction, which are essential in assessing the importance of the identified rules. By considering these metrics, we aim to emphasize the significance of the generated rules and minimize the possibility of errors in detecting heart disease.

In summary, this paper presents a novel framework for generating and evaluating association rules from medical data, specifically targeting heart disease detection. By integrating multiple statistical measures, our approach aims to uncover meaningful patterns that can significantly enhance the understanding, early detection, and treatment of heart disease. The ultimate goal is to provide a methodology that aids clinicians in making informed decisions, thereby improving patient outcomes and contributing to the broader field of cardiovascular research.

## Related work

In spite of the significant challenges presented by heart disease, which remains the leading cause of death worldwide, machine learning techniques have greatly assisted in the analysis of clinical data. These techniques make use of the vast amount of healthcare data that is readily available and have become powerful decision-making and forecasting tools.

Various studies have explored the potential of machine learning in predicting heart disease. In a study^26^, the Random Forest algorithm emerged as the most accurate method for predicting heart disease. Another study^27^ proposed an innovative approach that combines various features and classification techniques to enhance prediction accuracy. In a research^28^, machine learning techniques for heart disease prediction were reviewed, revealing a variety of data mining strategies with varying degrees of effectiveness and accuracy. Similarly, a study^29^ performed a comprehensive review of different machine learning techniques, including Artificial Neural Networks, Decision Trees, Fuzzy Logic, K-Nearest Neighbours, Naïve Bayes, and Support Vector Machines, in the context of heart disease prediction.

Furthermore, extensive research has been conducted to predict and evaluate the risk factors associated with heart disease. In a study^30^, various machine learning algorithms, such as logistic regression and KNN, were used to predict and classify patients with heart disease. Another study^31^ utilized the optimized LightGBM classifier with improved hyperparameters and a focal loss function optimized through OPTUNA. This model, evaluated on CVD data from the Framingham Heart Institute, achieved an AUC value of 97.8%, outperforming other comparative models in terms of accuracy.

A novel Recommendation System for CVD Prediction Using an IoT Network (DEEP-CARDIO) was proposed in another study^32^, offering prior diagnosis, treatment, and dietary recommendations for cardiac diseases. This system collects data from four biosensors (ECG, pressure, pulse, and glucose) and processes it using an Arduino controller. The BiGRU attention model diagnoses and classifies CVD into five categories, achieving an overall accuracy of 99.90%. Furthermore, the QMBC technique, which employs the Quine McCluskey method to derive the Minimum Boolean expression for the target feature, was introduced^33^. By combining predictions from seven classifiers, the ensemble model forms a comprehensive dataset to apply the minimum Boolean equation with an 80:20 train-to-test ratio. The proposed QMBC model demonstrates superior performance compared to current state-of-the-art models and previously suggested methods, indicating its potential for improved cardiovascular disease prediction.

Although many machine learning techniques have been proposed for the early detection and diagnosis of heart disease, clinicians often struggle to trust these models due to their lack of interpretability. This difficulty in understanding the basis for the predictions compromises the reliability and acceptance of these models. To address this issue, it is essential to focus on developing transparent and interpretable models that enable clinicians and patients to comprehend the underlying mechanisms and have confidence in the model’s predictions. Several studies have investigated the utilization of rule-based methods, particularly association rule mining, in the domain of heart disease detection.

A novel methodology and algorithm for mining distributed medical data sources using association rules, specifically focusing on predicting heart disease, was presented in a study^34^. Another study^35^ utilized association rule mining to uncover concealed patterns related to frequently occurring heart diseases within the Bangladeshi population. Associative classification mining was employed in another research^36^ to construct a classifier with prediction rules of high interestingness values for accurate heart disease prediction. An enhanced association rule mining approach for detecting coronary artery disease using a heart disease dataset was introduced in a study^37^.

While current methods focus on improving prediction accuracy and identifying factors that contribute to cardiac disease, several limitations persist. One significant challenge is managing unlabeled data, which is crucial for developing robust and comprehensive models. Additionally, these approaches often fail to explore the relationships between various symptoms and heart disease-causing factors, potentially overlooking critical indicators.

Many recent studies^38^ have generated rules based on frequent patterns, resulting in predictable and well-known outcomes. Despite their utility, these studies often produce an overwhelming number of rules, making analysis and interpretation costly. To address this limitation, we introduce a novel modeling approach designed to generate a limited number of insightful and interesting rules, enhancing both the efficiency and effectiveness of rule analysis.

Association rule mining, particularly for rare patterns, is essential yet challenging for making decisions about heart disease. In this work, we propose a novel method that not only identifies factors leading to heart disease but also uncovers patterns that may indicate future disease development. Our approach uses frequent patterns as a foundation for discovering interesting patterns associated with heart disease. We developed a model to identify these patterns and their potential to lead to heart disease when combined with specific risk factors.

## Dataset: heart disease

The purpose of this section is to provide a brief overview of the heart disease dataset utilized in our research. Our study aims to identify predictive factors that lead to heart disease and to analyze patterns among healthy patients who may develop heart disease in the future. The dataset used in this research was obtained from the well-established IEEE DataPort^39^.

In this study, several popular heart disease datasets are combined to create a comprehensive dataset that was previously unavailable. This new dataset comprises 1190 instances and 12 common features, making it the largest heart disease dataset currently available for research. The dataset was curated from five different sources: Cleveland, Hungarian, Swiss, Long Beach VA, and Statlog (Heart). By integrating these datasets into a single resource, we aim to facilitate the advancement of machine learning and data mining algorithms related to heart disease. This comprehensive dataset will enable researchers to develop more accurate and effective methods for detecting and preventing heart disease.

Tables 1 and 2 present the characteristics of the dataset. Table 1 summarizes several key characteristics, while Table 2 provides a description of the nominal attributes.

**Table 1:** Heart disease dataset characteristics.

S. No.	Attribute	Description	Unit	Data type
1	Age	Age	Years	Numeric
2	Sex	Gender	1 = Male, 0 = Female	Binary
3	Chest pain type	Type of chest pain	1, 2, 3, 4	Nominal
4	Resting blood pressure	Resting blood pressure	mm Hg	Numeric
5	Serum cholesterol	Serum cholesterol level	mg/dl	Numeric
6	Fasting blood sugar	Fasting blood sugar	1 = True, 0 = False (>>120 mg/dl)	Binary
7	Resting ECG results	Resting electrocardiogram results	0, 1, 2	Nominal
8	Maximum heart rate achieved	Maximum heart rate achieved	{min = 60, max = 202}	Numeric
9	Exercise induced angina	Exercise-induced angina	0 = No, 1 = Yes	Binary
10	Oldpeak	ST depression induced by exercise relative to rest	Depression	Numeric
11	Slope of the peak exercise ST segment	Slope of the peak exercise ST segment	1, 2, 3	Nominal
12	Class	Diagnosis of heart disease	0 = No, 1 = Yes	Binary

**Table 2:** Description of nominal attributes.

Attribute	Description
Sex	1 = Male
	0 = Female
Chest pain type	1: Typical angina
	2: Atypical angina
	3: Non-anginal pain
	4: Asymptomatic
Fasting blood sugar	(Fasting blood sugar $> 120$ mg/dl) 1 = True, 0 = False
Resting ECG results	0: Normal
	1: Having ST-T wave abnormality (> 0.05 mV)
	2: Showing left ventricular hypertrophy by Estes’ criteria
Exercise induced angina	1 = Yes
	0 = No
Slope of the peak exercise ST segment	1: Upsloping
	2: Flat
	3: Downsloping
Class	1 = Heart disease
	0 = Normal

To ensure the highest level of data quality and consistency, a rigorous preprocessing pipeline was developed, which included several crucial steps such as handling missing values and standardizing the representation of data. Our dataset did not contain any missing or null values, and a value of 0 was only found once in an instance where **St slope** was 0. As it did not contribute to pattern or rule generation, we removed it, resulting in a total of 1189 transactions. However, since our proposed work involves unsupervised techniques rather than classification tasks, it is crucial that we perform preprocessing and feature selection that are tailored to our objectives. These processes will be described in detail in the following sections.

## The proposed model: EPFHD-RARMING

**Figure 2:**
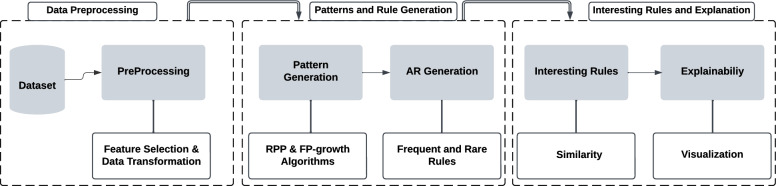
EPFHD-RARMING model for detecting heart disease risk factors.

Our proposed model, EPFHD-RARMING, builds upon previous work^40^ and aims to generate rules that facilitate the early detection of heart disease and predict the factors contributing to its development. This is achieved through a three-phase process, specifically designed as a case study for heart disease. Our approach allows for the identification of rare but significant associations that traditional methods often overlook, providing deeper insights into the factors leading to conditions like heart disease. The workflow of this model is illustrated in Fig. 2. Utilizing our method for enhancing rule-based machine learning in medical datasets, we developed and implemented the *Mine Interesting Rules* Algorithm 1. This algorithm systematically mines interesting rules from a dataset through three main phases, ensuring comprehensive analysis and interpretation.

**Algorithm 1:**
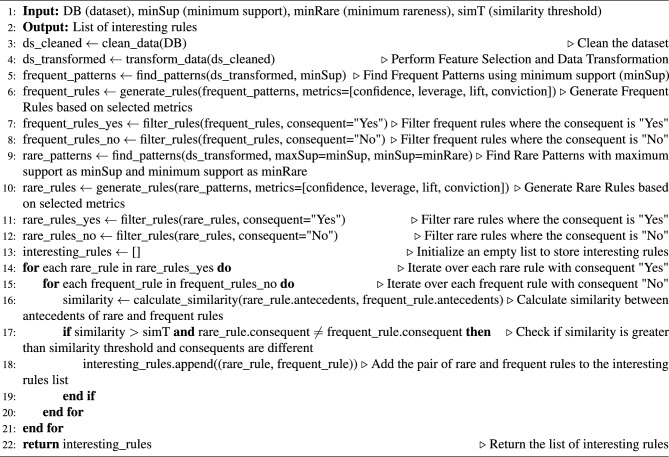
Mine interesting rules.

### Explanation of the algorithm

In this subsection, we explain the proposed algorithm and demonstrate its functionality. Algorithm 1 outlines a process for mining interesting rules from a dataset through three main phases.

#### Phase 1: Data preparation and cleaning

In the first phase, the dataset (*ds*) is prepared to handle missing values, outliers, and noise, followed by feature selection and data transformation to make it suitable for ARM. In lines 1–2, the algorithm starts by defining the inputs (*ds*, *minSup*, *minRare*, *simT*) and the expected output, which is a list of interesting rules. Here, *ds* stands for dataset, *minSup* for minimum support threshold, *minRare* for minimum rare support, and *simT* for similarity threshold.

In line 3, the dataset is cleaned to handle missing values, outliers, and noise, ensuring data quality for further analysis. In line 4, after data cleaning, the dataset undergoes feature selection and data transformation to prepare it for ARM, making the data suitable for pattern discovery.

#### Phase 2: Pattern discovery and rule extraction

From lines 5 to 12, the second phase involves pattern discovery and rule extraction. In line 5, frequent patterns are identified using a minimum support threshold (*minSup*). From lines 6 to 8, frequent rules are generated and filtered based on specified metrics and categorized into two kinds of rules based on their consequent values (“Yes” for heart disease and “No” for healthy). Similarly, rare patterns are found using both minimum support (*minSup*) and minimum rareness (*minRare*) thresholds, and corresponding rare rules are generated and filtered from lines 9 to 12.

#### Phase 3: Insightful rule identification and interpretation

In the final phase, from lines 13 to 22, interesting rules are identified by comparing rare rules with frequent ones. The similarity between the antecedents of rare rules (with a “Yes” consequent) and frequent rules (with a “No” consequent) is calculated. If the similarity exceeds a specified threshold (*simT*) and the consequences differ, the pair of rules is considered interesting and added to the list of interesting rules. The algorithm ultimately returns this list of interesting rules.

By following these detailed steps, the algorithm effectively cleans and transforms the data, discovers frequent and rare patterns, generates and filters rules, and identifies interesting rules for further analysis.

In the following subsections, a thorough explanation of each phase of the model will be provided, including the data preparation and transformation phase, Pattern discovery and rule extraction phase, and insightful rule identification and interpretation phase. The ultimate goal of this model is to provide a comprehensive understanding of its operation, with the aim of aiding in the early detection of heart disease and predicting the factors that contribute to its development.

### Data preparation and transformation phase

This section will concentrate on the preprocessing phase, which entails transforming the dataset from a supervised classification task to an unsupervised association rule mining task. The preprocessing phase is crucial in preparing the heart disease dataset for the mining process. We will examine in detail the two key steps of the preprocessing phase, as outlined in the initial phase of our workflow and described in lines 1–2 of the proposed Algorithm 1.

- Selection of features: In this stage, we employ various techniques to identify the most pertinent factors contributing to heart disease. This involves selecting a subset of the most informative features for the subsequent mining process. Appropriate feature selection can enhance the quality and efficiency of the mining process. Consequently, the feature selection process enables us to identify the most crucial attributes related to heart disease. It is a critical step in the knowledge discovery process that can help refine the understanding of heart disease and its associated factors.
- Dataset transformation: The transformation of the dataset for heart disease is crucial to ensure that it is suitable for mining association rules. The ideal format for mining association rules is a boolean transactional representation. This representation involves each instance being represented as a set of items, where each item represents a selected feature. The value of each item is either present (1) or absent (0). By undertaking this transformation, the dataset is prepared for further mining to generate association rules.

The following information provides a detailed description of the steps taken for the heart disease dataset that was utilized in this research:

#### Selection of features

The dataset for cardiovascular diseases contains 12 features, as illustrated in Table 1. To determine the essential features that contribute to cardiovascular disease, we have implemented a reliable feature selection process utilizing five distinct approaches. It is crucial to incorporate all relevant features that impact the heart disease to gain a comprehensive understanding of this condition. Through the following selection methods, we derived a final set of 10 features from the 12 features in the heart disease dataset. To choose the most crucial feature for the mining process, we employed the following Scikit-learn feature selection techniques:

- Feature selection using the chi-squared statistic.
- Feature selection using ANOVA F-value.
- Features selected through mutual information.
- Features selected via Recursive Feature Elimination with logistic regression.
- Features selected based on random forest feature importance.

**Figure 3:**
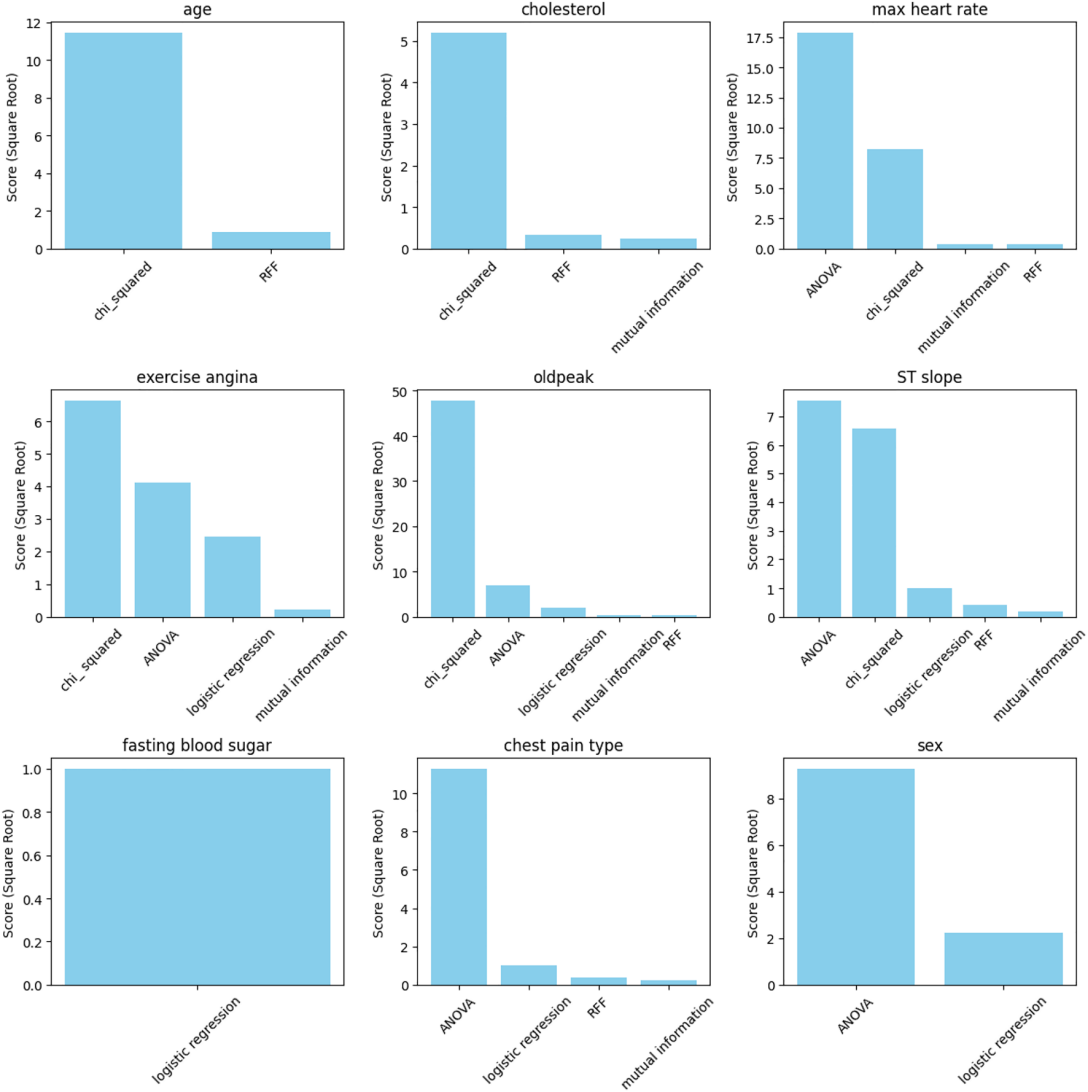
Key features contributing to the prediction of heart disease as identified by multiple feature selection methods.

Figure 3 illustrates the significance of the selected features using all the approaches employed in this paper. According to the graph, the features *’ST slope’* and *’oldpeak’* appear to have the greatest influence on the outcome variable, as they are included by all approaches. *’Max heart rate’*, *’exercise angina’*, and *’chest pain type’* occupy a secondary position in terms of importance, being favored by four out of five applied methods. The selection of *’cholesterol’* is made by three of the five methods, while the selection of *’age’* and *’sex’* is made by two methods, and the selection of *’fasting blood sugar’* is made by a single method.

To comprehensively address the majority of the important factors, all these features are incorporated into our approach. Therefore, the following features were chosen for this study: **{’ST slope’, ’age’, ’chest pain type’, ’cholesterol’, ’exercise angina’, ’fasting blood sugar’, ’max heart rate’, ’oldpeak’, and ’sex’}**. These features represent the union of the top features from each feature selection method. Additionally, the class feature *’target’* is included. As a result, 10 of the 12 features are utilized in our paper.

Undertaking extensive feature selection and concentrating our examination on the chosen features, we endeavor to gain a thorough understanding of the aspects that significantly influence the prevalence of heart disease.

#### Dataset transformation

To ensure the mining process is effective, it is essential to present all features in the dataset related to heart disease in a binary format. Four continuous attributes, namely ’age’, ’cholesterol’, ’max heart rate’, and ’oldpeak’, must be discretized to convert continuous data into discrete categories or bins. The process for discretizing these four features is as follows:

- The ’age’ feature is divided into three bins: ’young’, ’middle-aged’, and ’elderly’. The bin edges are specified as [0, 30, 60, np.inf], where ’np.inf’ represents infinity.
- The ’cholesterol’ feature is discretized into three bins: ’chollow’, ’cholnormal’, and ’cholhigh’. The bin edges are defined as [$-1$, 200, 240, np.inf].
- The ’max heart rate’ feature is discretized into three bins: ’heartratelow’, ’heartratenormal’, and ’heartratehigh’. The bin edges are specified as [0, 100, 160, np.inf].
- The ’oldpeak’ feature is discretized into three bins: ’oldpeaklow’, ’oldpeakmoderate’, and ’oldpeakhigh’. The bin edges are specified as [− np.inf, 1.0, 2.0, np.inf].

**Figure 4:**
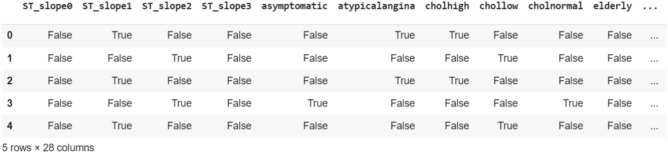
Dataset after preprocessing phase.

Transforming continuous variables into discrete categories streamlines data representation and expedites subsequent data analysis during the mining process. To prepare the dataset for analysis, it is crucial to convert the data into a binary format, such as [0, 1] or True/False. The TransactionEncoder() method is utilized for one-hot encoding, resulting in a final dataset consisting of 1189 rows and 28 dimensions. A representation of the first 5 rows of the preprocessed dataset is depicted in Fig. 4.

### Pattern discovery and rule extraction phase

The objective of this subsection is to detail the process of pattern and rule generation, a critical component of the proposed model, as depicted in lines 5–12 of the Algorithm 1. This phase is crucial for identifying and extracting meaningful relationships within the data, permitting the discovery of both frequent and rare patterns that contribute to the overall analysis.

#### Pattern generation

During the process of pattern discovery, we employ a formal technique to identify relevant patterns, which subsequently generate association rules. This process involves the exploration of both frequent and rare patterns present within the dataset. To accomplish this, we utilize specialized algorithms designed for each type of pattern. The FP-growth algorithm is employed to uncover frequent patterns^41^. This algorithm is well-known for its capacity to produce patterns that satisfy a specified support threshold. By using this algorithm, we are able to pinpoint patterns that frequently occur and hold significance within the dataset. These patterns represent commonly recognized phenomena and expected information.

In addition to frequent patterns, we also uncover rare patterns, which provide distinctive insights. To achieve this, we utilize the Rare Pre-Post (RPP) algorithm^42^. This algorithm enables us to identify rare patterns within the dataset that occur infrequently but are intriguing and valuable.

By merging FP-growth for frequent pattern mining with RPP for rare pattern mining, we can generate a comprehensive set of patterns. This approach allows us to produce a complete set of association rules, encompassing both rare and frequent rules. As a result, we acquire meaningful insights and extract valuable knowledge from the data.

#### Rule generation

After analyzing frequent and rare patterns, we can derive association rules. These rules offer valuable insights into the relationships and dependencies between various items and attributes in a dataset. By examining these rules, we can gain a thorough understanding of the underlying patterns and associations in the data.

Our model generates two types of rules: frequent rules and rare rules. Frequent rules represent significant associations within the dataset, determined by their high support and satisfaction of multiple statistical metrics. In this study, we concentrate on rules where the consequent signifies healthy patients without heart disease. These frequent rules expose health attributes or factors connected to good health and the absence of heart disease.

On the other hand, rare patterns lead to rules with low support but significant statistical relevance based on the metrics we applied. The inspection of these rare rules provides insights into unique factors associated with heart disease. In this study, rare rules are used to represent non-healthy patients, particularly those with heart disease.

Analyzing both frequent and rare rules provides a comprehensive understanding of the associations and dependencies present in the data, enabling the determination of which attributes or factors contribute to good health and which indicate heart disease. This dual analysis allows for the extraction of valuable knowledge from the data and the making of informed decisions based on identified patterns.

### Insightful rule identification and interpretation

The third and final phase of our proposed model, detailed in lines 13–22 of Algorithm 1, focuses on generating and interpreting interesting rules. This critical phase aims to identify the features that contribute to heart disease and uncover meaningful rules. By establishing a set of beliefs-frequent rules that represent healthy patients without heart disease-we aim to identify individuals with heart disease whose characteristics differ subtly from those of healthy individuals. This approach allows us to highlight the distinct features associated with heart disease, providing valuable insights for early detection and targeted interventions.

#### Interesting rules

By identifying rare rules with low support that satisfy all statistical metrics based on the background information provided, we can further refine the set of interesting association rules. In this study, we aim to identify those rare rules that deviate from the common (frequent) rules representing healthy individuals. To determine the interestingness and unexpected nature of these rules, we consider the following factors:

- **Similarity of antecedents**: We assess the similarity between the antecedents of rare rules and frequent rules using a similarity measure, such as the Jaccard similarity approach.
- **Contrasting consequences**: We evaluate whether the consequences of the rare rules contrast with those of the frequent rules. For a rule to be considered interesting, the consequences should oppose each other.
- **Low support**: The rare rule (Rrule) must meet all predefined metrics, particularly having low support. This indicates its deviation from the normal (frequent) rule (Frule).

By incorporating these criteria, we can filter and prioritize rare rules that exhibit low support, deviate from normal patterns, have similar antecedents, and contrasting consequences with frequent rules. These refined rules offer valuable insights into the underlying patterns and deviations within the dataset, enhancing our understanding of exceptional cases and unexpected associations.

#### Explainability

The utilization of association rule mining, a data mining approach based on rules, is of paramount importance due to its interpretability and ease of comprehension. In our research, we have presented a summary of the noteworthy rules generated by our model, emphasizing the elements that contribute to heart disease. To increase the clarity and understanding of these association rules, our model is supported by extensive documentation, including tables, examples, and illustrations.

Through the identification of the factors in the rare rules that differ from our established beliefs, represented by the frequent rules, we are able to gain deeper insights into the specific factors contributing to heart disease. This knowledge is invaluable for deciphering the underlying causes of heart disease and can play a significant role in its prevention and treatment.

## Experimental results

In this section, we present the outcomes of our proposed model, EPFHD-RARMING, which aims to generate concise and valuable rules for heart disease prediction. We provide a comprehensive explanation of the results, demonstrating the effectiveness and efficiency of our model in producing these desired rules without an excessive number. Through extensive analysis, we emphasize the significance of the generated rules and their potential implications in the field of heart disease prediction. The experimental results section details the findings and insights derived from our in-depth experiments and analyses, with a focus on the important rules, factors, and relationships between heart disease risk factors observed by our model. In the subsequent subsections, we elaborate on the results obtained through the use of the proposed approach, EPFHD-RARMING.

### Experimental setup

In this paper, the experiments were conducted on Google Colab using the following commonly used parameters and constraints. While mining both frequent and rare patterns for pattern generation, it is essential to adhere to the following constraints.

- To obtain frequent patterns, a minimum support threshold, *minSup*, of 0.01 was established in order to identify frequent patterns. This signifies that a pattern is classified as frequent only when it occurs in no fewer than 0.01 of the total instances within the dataset.
- Rare patterns are identified by focusing on patterns with support below this minimum support, *minSup*, and above the minimum support, *minRare*, of 0.001, denoted as $minRare = 0.001$. Therefore, we aim to identify rare patterns with support less than *minSup* and support equal to or greater than *minRare*.

With regard to rule generation, it is necessary to adhere to several conditions to determine the criteria for compelling rules within the proposed model. These conditions are applicable to both frequent and rare rules. Thus, only rules that meet these stringent criteria are recognized in our proposed model.

**Minimum support of rules**: Frequent rules must have a minimum support of *minSup*. In same way, for rare rules, we consider rules with support less than *minSup*, but still exceeding *minRare*.

**Metric requirements**: To be considered a strong rule, whether frequent or infrequent, the below popular metrics must be met.

- **Confidence**: Confidence score must exceed 0.80.
- **Lift**: Lift must be greater than 1.
- **Leverage**: Leverage should be greater than 0.
- **Conviction**: Conviction should be greater than 1.

In addition, for brevity, we replace the names of columns with abbreviations, as shown in Table 3. By equalizing full column names with their abbreviated counterparts (e.g., ’asymptomatic’ to ’asym’ and ’heart_disease’ to ’yes’), the mapping provides clarity and brevity in data representation.

**Table 3:** Column name mapping.

No	Original name	Shortened name	No	Original name	Shortened name
1	Asymptomatic	asym	15	heart_rate_high	hrhigh
2	Atypical_angina	atangina	16	heart_rate_low	hrlow
3	Cholhigh	hcol	17	heart_rate_normal	hrnoraml
4	Chollow	lcol	18	middle-aged	maged
5	Cholnormal	ncol	19	non_anginal_pain	napain
6	Downsloping	dsloping	20	oldpeakhigh	peakhigh
7	Elderly	elderly	21	oldpeaklow	peaklow
8	Exercise_angina0	exangina0	22	oldpeakmoderate	peakmoderate
9	Exercise_angina1	exangina1	23	typical_angina	tangina
10	Fasting_blood_sugar0	fbsugar0	24	upsloping	usloping
11	Fasting_blood_sugar1	fbsugar1	25	young	young
12	Female	F	26	heart_disease	yes
13	Man	M	27	no_heart_disease	no
14	Flat	flat			

### Patterns generation

**Figure 5:**
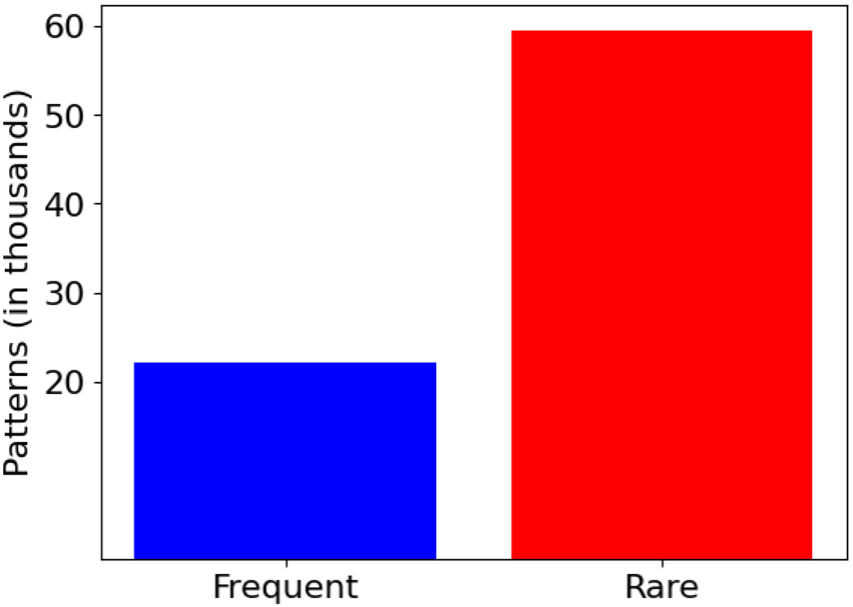
A comparison of the number of frequent and rare patterns generated from the heart disease dataset.

To identify frequent patterns, we employ the FP-growth algorithm^41^. Additionally, we use the RPP algorithm^42^ to detect rare patterns that may result in unexpected outcomes. Figure 5 illustrates the results of our case study on heart disease. By applying very low support levels, the graph reveals a substantial number of rare patterns. In this phase of pattern generation, we identified a total of 81,632 patterns, of which 22,178 are frequent and 59,454 are rare.

After generating both frequent and rare patterns, we proceed to derive association rules. Our approach emphasizes identifying the most valuable and significant rules while filtering out the majority of less-relevant rules derived from rare patterns. The subsequent section will detail the process of extracting these intriguing and insightful rules from the comprehensive set of patterns.

### Rule generation

Following the generation of frequent and rare patterns as depicted in Fig. 5, the next step involves formulating an exhaustive set of association rules. As shown in Fig. 1, a significant number of rules, 55,307 in total are generated from the frequent patterns, where the antecedent support meets or exceeds the specified minimum support threshold ($minSup = 0.01$). Additionally, a much larger number of rules, totaling 389,531, are derived from the rare patterns, where their support falls below the *minSup* threshold. This substantial quantity of rules underscores a critical limitation within the domain of association rule mining and highlights the need for a methodology that facilitates the efficient identification of insightful rules.

The primary goal and challenge of this study are to develop a methodology that can identify rules revealing factors contributing to heart disease detection. Specifically, we focus on rules that are most relevant to this objective.

**Figure 6:**
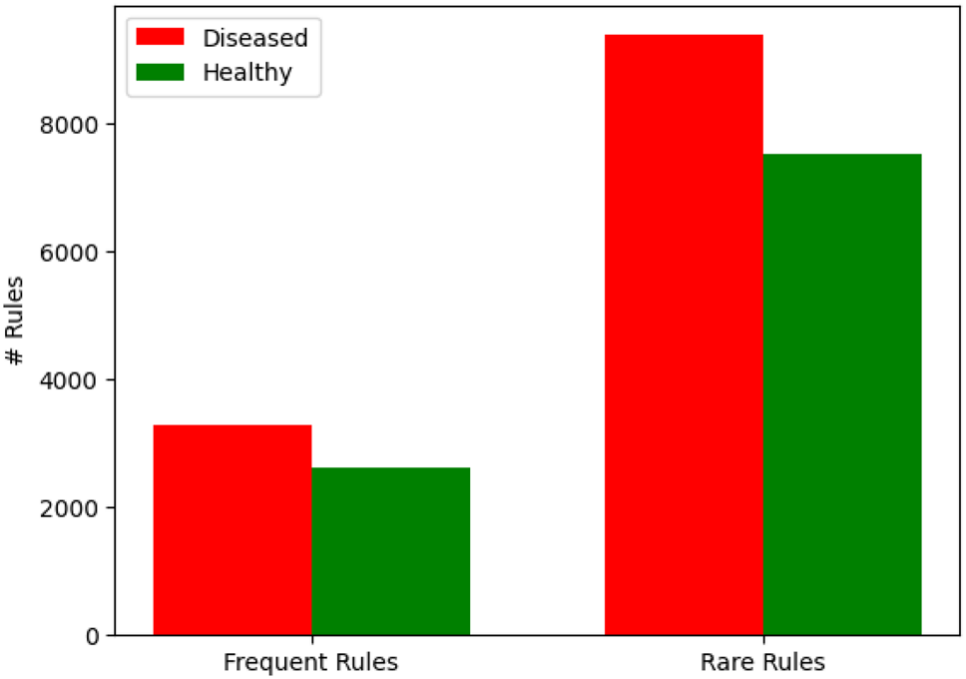
A comparison of the number of frequent and rare rules generated from the heart disease dataset.

To mitigate the overwhelming growth of rules and address the aforementioned challenge, we undertook an extensive exploration of rule generation. To enhance understanding, the rules have been organized based on their outcomes, specifically distinguishing between those that indicate the presence or absence of heart disease. Consequently, our focus is directed solely towards rules that relate to the presence or absence of heart disease, aligning with our primary objective. As shown in Fig. 6, these rules can be classified into four distinct categories:

- Frequent rules leading to the occurrence of heart disease.
- Frequent rules that emphasize health in the absence of heart disease.
- Rare rules indicating the presence of heart disease.
- Rare rules suggesting the absence of heart disease.

It is essential to highlight that all the rules being considered are considered dependable, as they fulfill all the necessary requirements outlined in our experimental framework. As a result, the rules can be classified into the following four categories:

- **Type 1**: There are 2624 rules that are frequent and have the consequence “no heart disease”. Here is an example for such kind of rules: {**’maged’, ’fbsugar0’, ’usloping’, ’peaklow’** $\Rightarrow$ **’No’**}. The rule’s various evaluation metrics are as follows: **support (0.24), confidence (0.86), lift (1.81), leverage (0.11), and conviction (3.73)**.
- **Type 2**: There are 3293 frequent rules with “heart disease” as their consequent. Here is an example of such kind of rule: {**hrnoraml’, ’exangina1’, ’asym’, ’M’** $\Rightarrow$ **’Yes’**}. The rule’s various evaluation metrics are as follows: **support (0.21), confidence (0.92), lift (1.7), leverage (0.08), conviction (6.02)**.
- **Type 3**: A total of 7530 rules are rare and have “no heart disease” as an outcome. For example, the rule: **{’fbsugar0’, ’tangina’, ’hrnoraml’, ’exangina0’, ’dsloping’, ’M’}** $\Rightarrow$ **’No’** indicates there is no presence of heart disease. The rule’s various evaluation metrics are as follows: **support (0.001), confidence (1.0), lift (2.11), leverage (0.0008), conviction (Infinity)**.
- **Type 4**: A total of 9381 rare rules indicate the presence of “heart disease”. In the case of this type of rule, for example, {**’asym’, ’exangina1’, ’hrnoraml’, ’flat’, ’ncol’, ’elderly’, ’M’** $\Rightarrow$ **’Yes’**}. The rule’s various evaluation metrics are as follows: **support (0.009), confidence (1.0), lift (1.89), leverage (0.004), conviction (Infinity) **.

#### Type 1 and 2 (frequent rules)

As shown in Fig. 6, Type 1 rules consist of 2624 frequent rules associated with the consequence “no heart disease.” These rules represent healthy patients and indicate that the exhibited symptoms do not suggest the presence of heart disease. Conversely, Type 2 rules encompass frequent rules with high support that represent patients with heart disease. These rules reflect a well-established phenomenon where frequent rules exhibit a high level of support and align with specific expectations.

The insights gained from these rule types are widely recognized and can be readily interpreted by domain experts. Numerous studies have extensively examined this category of rules^38^, leading us to regard them as a set of prevailing beliefs, as they encapsulate the most commonly occurring patterns.

Our analysis focuses on Type 1 rules, which we use as the foundation for identifying unexpected and intriguing rules. We elaborate on this endeavor in the following section, “Interesting Rules,” which constitutes the principal contribution of our novel model, EPFHD-RARMING.

#### Type 3 and 4 (rare rules)

Typically, traditional methodologies primarily concentrate on the analysis of rules that fall under categories 1 and 2, while rules of types 3 and 4 are often not given due consideration. However, it is imperative to acknowledge that rules within categories 3 and 4 possess the potential to yield more insightful and valuable findings. Consequently, identifying noteworthy rules among this extensive collection poses a significant challenge, particularly when attempting to discover unexpected and substantial rules connected to the incidence of heart disease.

As part of our research endeavor, we undertake a comprehensive examination of these commonly overlooked rules. Our primary focus is on type 4 rules, which comprise 9381 rare rules that indicate the presence of “heart disease.” By analyzing these rules, we aim to uncover factors that contribute to the development of heart disease. Conversely, we choose to exclude the rules of type 3, which typically indicate healthy patients and exhibit low levels of support in our dataset. An extensive analysis of these rules would incur excessive costs without yielding significant insights into our primary objective: identifying patients with heart disease. For further information, these categories are depicted in Fig. 6 as rare rules.

### Interesting rules

It is the primary objective of this subsection to identify and investigate the unusual or uncommon rules that differ from those observed in cases without cardiac conditions. The interesting aspect of these rules lies in their similarity to the factors and their consequent contrast with one another, resulting in unexpected outcomes. Consequently, we analyzed both type 4 rules (the rare rules indicating heart disease) and type 1 rules (the frequent rules indicating healthy patients).

To determine the interesting rules, we employed objective metrics such as lift, confidence, leverage, and conviction, as defined in Definitions 2 and 3. These rules, whether frequent or rare, must satisfy these metrics to be considered strong and demonstrate their objective interest.

Moreover, we explored rare rules that deviate from the normal rules (i.e., frequent rules without heart disease) due to symptoms that reduce the support of the rules. To identify such rules, we utilized the Jaccard metric and set the similarity threshold at 0.80. This allowed our study to identify patterns associated with the absence of heart disease that become rare in the presence of heart disease when another factor is introduced. Consequently, we identified a total of 163 interesting rules using our proposed model. Analyzing these rules can provide valuable insights for medical experts, particularly in identifying symptoms that may be indicative of heart disease.

Our model, EPFHD-RARMING, was successful in extracting 163 relevant rules from a vast number of rules. These rules can provide valuable insights to medical experts in their investigation of symptoms that may indicate cardiovascular disease.

Let us analyze two specific rules, denoted as “frequent” and “rare.” The first rule, represented as **’heartrate = normal’, ’oldpeak = high’, ’exercise angina = 0’, ’fasting blood sugar = 0’, ’cholesterol = high’, ’sex = female’ ==> ’Yes’ (heart disease)**, is classified as a **rare rule**. Conversely, the second rule, expressed as **’heartrate = normal’, ’exercise angina = 0’, ’fasting blood sugar = 0’, ’cholesterol = high’, ’sex= female’ ==> ’No’ (no heart disease)**, is categorized as a **frequent rule**. These two rules demonstrate a substantial degree of similarity, quantified at 0.83. This similarity indicates that when a seemingly healthy patient exhibits specific characteristics, including normal heart rate, absence of exercise-induced angina, normal fasting blood sugar levels, high cholesterol levels, and female gender, a flag is raised suggesting a potential risk of heart disease, particularly if their oldpeak value is high. In other words, it is important to note that a patient may develop heart disease if their oldpeak value becomes high while exhibiting the symptoms indicated in the frequent rule.

**Figure 7:**
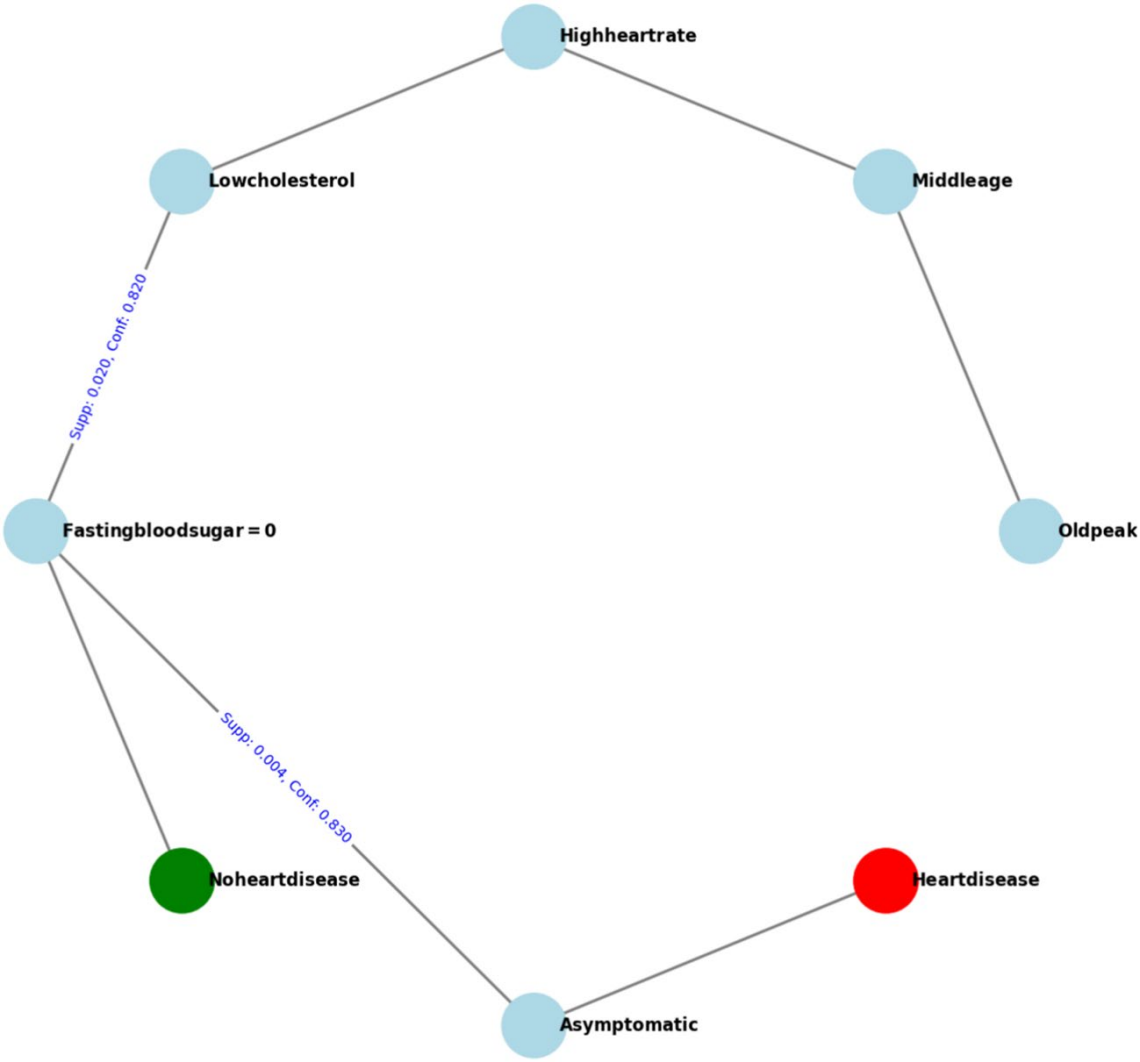
Visualization of the rules highlighting the significance of the ’asymptomatic’ feature in altering predictions from ’No’ to ’Yes’ for heart disease. The graph illustrates how our proposed model identifies interesting rare rules by showing changes in support and confidence when the ’asymptomatic’ condition is added. This example demonstrates the primary results of our work, emphasizing the importance of considering rare but meaningful rules in heart disease detection.

The visualization presented in Fig. 7 illustrates two critical rules derived from our heart disease dataset. These rules highlight the importance of identifying rare but significant patterns that can drastically alter prediction outcomes. The first rule, with a consequent “No heart disease,” has antecedents that include “Oldpeak = low,” “Middle age,” “High heart rate,” “Low cholesterol,” and “Fasting blood sugar = 0.” This rule has a support value of 0.02 and a confidence of 0.82, indicating that it is relatively common and reliable in predicting the absence of heart disease under these conditions.

The second rule, which includes the additional antecedent “Asymptomatic,” changes the prediction to “Heart disease.” Despite having a lower support value of 0.004, this rule boasts a higher confidence of 0.83. The transformation from a “No heart disease” to a “Heart disease” consequent upon adding the “Asymptomatic” condition underscores the critical nature of this rare pattern. The similarity in antecedents between these two rules, differing only by the presence of “Asymptomatic,” makes the second rule particularly intriguing and significant for heart disease detection.

This analysis highlights how our proposed model effectively identifies interesting rare rules by examining this example, which demonstrates the primary results of our work. By focusing on such rare but valuable rules, healthcare professionals can better identify and manage patients who might otherwise be overlooked due to the rarity of these conditions. This approach not only enhances the accuracy of heart disease predictions but also contributes to a more nuanced understanding of the various factors involved. The “Asymptomatic” feature, when combined with other symptoms, can change the risk assessment from no heart disease to high risk, emphasizing its role in medical diagnostics.

### Explanation and interpretation of interesting rules

**Figure 8:**
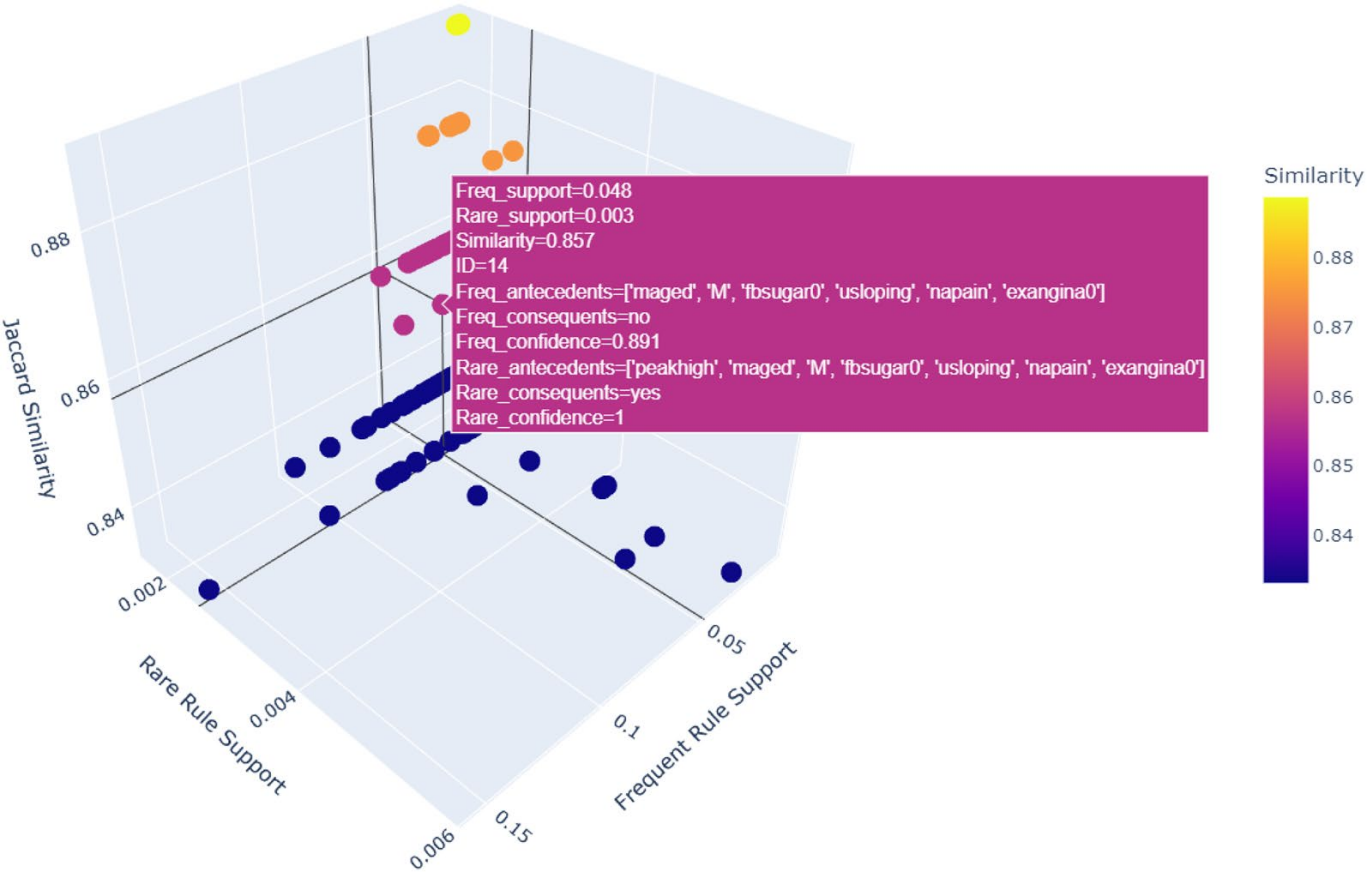
163 interesting rules plotted in 3D.

The purpose of this section is to provide a thorough and comprehensive explanation of the generated intriguing rules, ensuring that they are clearly communicated and understood by the end user. A total of 163 interesting rules have been identified by our model, and their visual representation can be found in Fig. 8. It is worth noting that the graph indicates a high similarity between frequent and rare rules, with a similarity score exceeding 0.80. Our focus is on the rare rules that deviate from the frequent rules by introducing additional symptoms, resulting in the formation of new rules with lower support but yielding more unexpected insights. For example, the labeled rules in the graph showcase both the frequent rule and the rare rules that diverge from it. Figure 8 visualizes the relationship between frequent rules and rare rules in terms of their support and similarity. The plot uses three dimensions to represent key metrics:

- **X-axis (frequent rule support):** This axis represents the support values of frequent rules, indicating how often these rules occur within the dataset.
- **Y-axis (rare rule support):** This axis represents the support values of rare rules, showing how often these less common rules appear within the dataset.
- **Z-axis (Jaccard similarity)** This axis represents the Jaccard similarity between the antecedents of frequent and rare rules. A higher similarity value indicates a greater overlap between the sets of conditions that define the rules.

The points in the plot are color-coded based on their similarity values, with the color bar on the side providing a reference for the similarity scale. The interactive nature of the plot allows users to hover over individual points to see detailed information, including the ID of the rule pair, the antecedents, consequents, support, and confidence of both frequent and rare rules, and the similarity value for each pair of rules.

In the highlighted example, the point represents a pair of rules with the following details:

- **Frequent rule:** {Antecedents: ’Middle age’, ’Male’, ’Fast blood suger =0, ’Upsloping ST slope’, ’Non-anginal pain’, ’Exercise-induced angina = 0’}, Consequent: ’No heart disease’, Support: 0.048, and Confidence: 0.89.
- **Rare rule:** {Antecedents: ’Middle age’, ’Male’, ’Fast blood suger =0, ’Upsloping ST slope’, ’Non-anginal pain’, ’Exercise-induced angina = 0’, ’OldPeak high’}, Consequent: ’heart disease’, Support: 0.003, and Confidence: 1.
- **Similarity (Jaccard):** 0.857.

This specific pair of rules is significant because the addition of the antecedent ’OldPeak High’ in the rare rule changes the consequent from ’No heart disease’ to ’Heart disease’. Despite the rare rule having a much lower support value, the high Jaccard similarity (0.86) with the frequent rule indicates that the conditions for both rules are very similar. This insight is crucial as it highlights how a slight change in conditions can alter the outcome, emphasizing the importance of considering rare rules in heart disease prediction and diagnosis. The confidence levels of both rules also suggest their reliability, making them valuable for further analysis and application in medical diagnostics.

Table 4 presents the most interesting rules based on their similarity measure. It is important to note that all these rules are frequent and correspond to healthy patients, becoming rare and indicative of heart disease when an additional symptom is included. A possible explanation for the occurrence of interesting rare rules in the dataset is that adding another factor or symptom to frequent rules reduces their support and makes them rare.

**Table 4:** Top 10 most interesting rare rules that.

No	Uncommon rules (heart disease)
1	{’peaklow’, ’maged’, ’hrhigh’, ’M’, ’lcol’, ’fbsugar0’, ’usloping’, ’exangina0’, **’asym’** }
2	{’ncol’, ’maged’, ’hrhigh’, ’fbsugar0’, ’usloping’, ’napain’, ’exangina0’, **’peakhigh’**}
3	{’hrnoraml’, ’peaklow’, ’maged’, ’lcol’, ’fbsugar0’, ’napain’, ’exangina0’, **’flat’**}
4	{’atangina’, ’hcol’, ’maged’, ’F’, ’usloping’, ’hrnoraml’, ’exangina0’, **’fbsugar1’**}
5	{’hrnoraml’, ’peaklow’, ’maged’, ’M’, ’lcol’, ’napain’, ’exangina0’, **’flat’**}
6	{’atangina’, ’hcol’, ’peaklow’, ’maged’, ’F’, ’usloping’, ’exangina0’, **’fbsugar1’**}
7	{’maged’, ’hrhigh’, ’M’, ’fbsugar0’, ’usloping’, ’napain’, ’exangina0’, **’peakhigh’**}
8	{’atangina’, ’hcol’, ’peaklow’, ’maged’, ’F’, ’fbsugar0’, ’hrnoraml’, **’flat’**}
9	{’peaklow’, ’maged’, ’hrhigh’, ’M’, ’lcol’, ’usloping’, ’exangina0’, **’asym’**}
10	{’atangina’, ’hcol’, ’peaklow’, ’maged’, ’F’, ’hrnoraml’, ’exangina0’, **’fbsugar1’**}

Let us take rule number 7 in Table 4 to illustrate how interesting rules are generated. In the absence of the red symptom **oldpeak with a high value**, the frequent rule with the factors **’middle-aged’, ’high heart rate’, ’male’, ’fasting blood sugar = 0’, ’upsloping ST slope’, ’no exercise-induced angina’ ==> ’no heart disease’** suggests that individuals with these factors are generally free from heart disease. However, when a new rule, a rare one, is formed by including a high ’oldpeak’ value, the generated rule **’high oldpeak’, ’middle-aged’, ’high heart rate’, ’male’, ’fasting blood sugar = 0’, ’upsloping ST slope’, ’no exercise-induced angina’ ==> ’heart disease’** identifies patients at risk of heart disease. While the support of the frequent rule is 0.06 out of 1189, indicating that approximately 71 patients with these factors are healthy, the support of this new rare rule decreases to 0.002, implying that only 2 patients with these factors actually have heart disease.

The following section provides an in-depth analysis of the factors contributing to the development of heart disease. Our findings underscore the significance of these factors and their impact on the prediction of heart disease, further validating our proposed model, EPFHD-RARMING. This analysis not only offers deeper insights into the relationship between these factors and heart disease but also enhances the interpretability and explainability of our findings.

It is imperative to emphasize that the rules outlined in Tables 5, 6, 7, 8, 9 and 10 pertain to healthy individuals, as they represent the most frequent rules associated with no heart disease (set of beliefs) and high support when the crucial “red feature” is absent. This “red feature” serves as the pivotal element that transforms these common rules—characterized by their high support and absence of cardiac disease—into uncommon rules with reduced support when cardiac disease is present. In the following subsections, we will provide a thorough examination of these contributing factors.

**Table 5:** Top 10 rare heart disease rules with high oldpeak values.

No	Uncommon rules (heart disease)
1	$\{$’ncol’, ’maged’, ’hrhigh’, ’fbsugar0’, ’usloping’, ’napain’, ’exangina0’, **’peakhigh’** $\}$
2	$\{$’maged’, ’hrhigh’, ’M’, ’fbsugar0’, ’usloping’, ’napain’, ’exangina0’, **’peakhigh’** $\}$
3	$\{$’ncol’, ’maged’, ’M’, ’fbsugar0’, ’usloping’, ’napain’, ’exangina0’, **’peakhigh’** $\}$
4	$\{$’ncol’, ’M’, ’fbsugar0’, ’usloping’, ’napain’, ’exangina0’, **’peakhigh’** $\}$
5	$\{$’maged’, ’hrhigh’, ’M’, ’usloping’, ’napain’, ’exangina0’, **’peakhigh’** $\}$
6	$\{$’maged’, ’hrhigh’, ’M’, ’fbsugar0’, ’usloping’, ’exangina0’, **’peakhigh’** $\}$
7	$\{$’maged’, ’hrhigh’, ’M’, ’fbsugar0’, ’usloping’, ’napain’, **’peakhigh’** $\}$
8	$\{$’maged’, ’M’, ’lcol’, ’usloping’, ’napain’, ’exangina0’, **’peakhigh’** $\}$
9	$\{$’ncol’, ’maged’, ’hrhigh’, ’M’, ’usloping’, ’napain’, **’peakhigh’** $\}$
10	$\{$’maged’, ’M’, ’fbsugar0’, ’usloping’, ’ncol’, ’exangina0’, **’peakhigh’** $\}$

**Table 6:** Top 10 rare heart disease rules: peak exercise ST segment (ST slope) with flat values.

No	Uncommon rules (heart disease)
1	$\{$’hrnoraml’, ’peaklow’, ’maged’, ’lcol’, ’fbsugar0’, ’napain’, ’exangina0’, **’flat’**$\}$
2	$\{$’hrnoraml’, ’peaklow’, ’maged’, ’M’, ’lcol’, ’napain’, ’exangina0’, **’flat’**$\}$
3	$\{$’atangina’, ’hcol’, ’peaklow’, ’maged’, ’F’, ’fbsugar0’, ’hrnoraml’, **’flat’**$\}$
4	$\{$’atangina’, ’hcol’, ’peaklow’, ’maged’, ’M’, ’fbsugar0’, ’hrnoram’, **’flat’**$\}$
5	$\{$’atangina’, ’hcol’, ’peaklow’, ’maged’, ’fbsugar0’, ’hrnoraml’, ’exangina0’, **’flat’**$\}$
6	$\{$’atangina’, ’hcol’, ’peaklow’, ’M’, ’hrnoraml’, ’exangina0’, **’flat’**$\}$
7	$\{$’peaklow’, ’M’, ’lcol’, ’napain’, ’hrnoraml’, ’exangina0’, **’flat’**$\}$
8	$\{$’atangina’, ’hcol’, ’peaklow’, ’F’, ’fbsugar0’, ’hrnoraml’, **’flat’**$\}$
9	$\{$’atangina’, ’hcol’, ’peaklow’, ’maged’, ’hrnoraml’, ’exangina0’, **’flat’**$\}$
10	$\{$’atangina’, ’hcol’, ’peaklow’, ’maged’, ’fbsugar0’, ’hrnoraml’, **’flat’**$\}$

**Table 7:** Top 10 Rare heart disease rules: chest pain type as asymptomatic.

No	Uncommon rules (heart disease)
1	$\{$’peaklow’, ’maged’, ’hrhigh’, ’M’, ’lcol’, ’fbsugar0’, ’usloping’, ’exangina0’, **’asym’**$\}$
2	$\{$’peaklow’, ’maged’, ’hrhigh’, ’M’, ’lcol’, ’usloping’, ’exangina0’, **’asym’**$\}$
3	$\{$’maged’, ’hrhigh’, ’M’, ’lcol’, ’fbsugar0’, ’usloping’, ’exangina0’, **’asym’**$\}$
4	$\{$’hcol’, ’peaklow’, ’elderly’, ’F’, ’fbsugar0’, ’usloping’, ’exangina0’, **’asym’**$\}$
5	$\{$’peaklow’, ’hrhigh’, ’M’, ’lcol’, ’fbsugar0’, ’usloping’, ’exangina0’, **’asym’**$\}$
6	$\{$’hrhigh’, ’M’, ’lcol’, ’fbsugar0’, ’usloping’, ’exangina0’, **’asym’**$\}$
7	$\{$’peaklow’, ’hrhigh’, ’M’, ’lcol’, ’usloping’, ’exangina0’, **’asym’**$\}$
8	$\{$’maged’, ’hrhigh’, ’M’, ’fbsugar1’, ’usloping’, ’exangina0’, **’asym’**$\}$
9	$\{$’hcol’, ’elderly’, ’F’, ’fbsugar0’, ’usloping’, ’exangina0’, **’asym’**$\}$
10	$\{$’hcol’, ’peaklow’, ’elderly’, ’F’, ’fbsugar0’, ’exangina0’, **’asym’**$\}$

**Table 8:** Rare heart disease rules: max heart rate as low.

No	Uncommon rules (heart disease)
1	$\{$’hcol’, ’elderly’, ’F’, ’fbsugar0’, ’napain’, ’exangina0’, **’hrlow’**$\}$
2	$\{$’peakmoderate’, ’elderly’, ’F’, ’fbsugar0’, ’flat’, ’exangina0’, **’hrlow’**$\}$
3	$\{$’F’, ’fbsugar0’, ’flat’, ’napain’, ’exangina0’, **’hrlow’**$\}$
4	$\{$’peakmoderate’, ’F’, ’fbsugar0’, ’flat’, ’exangina0’, **’hrlow’**$\}$
5	$\{$peakmoderate’, ’hcol’, ’F’, ’fbsugar0’, ’exangina0’, **’hrlow’**$\}$
6	$\{$’peakmoderate’, ’F’, ’fbsugar0’, ’napain’, ’exangina0’, **’hrlow’**$\}$
7	$\{$’hcol’, ’F’, ’fbsugar0’, ’napain’, ’exangina0’, **’hrlow’**$\}$
8	$\{$’hcol’, ’elderly’, ’F’, ’fbsugar0’, ’napain’, **’hrlow’**$\}$
9	$\{$’hcol’, ’elderly’, ’F’, ’napain’, ’exangina0’, **’hrlow’**$\}$
10	$\{$’peaklow’, ’maged’, ’fbsugar1’, ’ncol’, ’exangina0’, **’hrlow’**$\}$
11	$\{$’elderly’, ’F’, ’fbsugar0’, ’napain’, ’exangina0’, **’hrlow’**$\}$
12	$\{$’peakmoderate’, ’elderly’, ’F’, ’fbsugar0’, ’exangina0’, **’hrlow’**$\}$

**Table 9:** Rare heart disease rules: exercise-induced angina is present (as indicated by a value of 1).

No	Uncommon rules (heart disease)
1	$\{$’hcol’, ’peaklow’, ’maged’, ’fbsugar0’, ’usloping’, ’hrnoraml’, **’exangina1’**$\}$
2	$\{$’peakmoderate’, ’elderly’, ’F’, ’fbsugar0’, ’flat’, ’hrnoraml’, **’exangina1’**$\}$
3	$\{$’atangina’, ’hcol’, ’peaklow’, ’maged’, ’fbsugar0’, ’hrnoraml’, **’exangina1’**$\}$
4	$\{$’peaklow’, ’M’, ’elderly’, ’fbsugar0’, ’usloping’, ’ncol’, **’exangina1’**$\}$
5	$\{$’peaklow’, ’maged’, ’hrhigh’, ’lcol’, ’fbsugar0’, ’usloping’, **’exangina1’**$\}$
6	$\{$’maged’, ’F’, ’fbsugar0’, ’flat’, ’napain’, **’exangina1’**$\}$
7	$\{$’peakmoderate’, ’elderly’, ’F’, ’fbsugar0’, ’hrnoraml’, **’exangina1’**$\}$
8	$\{$’peaklow’, ’M’, ’elderly’, ’usloping’, ’ncol’, **’exangina1’**$\}$
9	$\{$’peaklow’, ’elderly’, ’fbsugar0’, ’usloping’, ’ncol’, **’exangina1’**$\}$
10	$\{$’peaklow’, ’maged’, ’hrhigh’, ’lcol’, ’fbsugar0’, **’exangina1’**$\}$
11	$\{$’peaklow’, ’hrhigh’, ’lcol’, ’fbsugar0’, ’usloping’, **’exangina1’**$\}$
12	$\{$’maged’, ’M’, ’fbsugar1’, ’usloping’, ’ncol’, **’exangina1’**$\}$
13	$\{$’peaklow’, ’maged’, ’hrhigh’, ’lcol’, ’usloping’ ,**’exangina1’**$\}$

**Table 10:** Rare heart disease rules: fast blood suger is present (as indicated by a value of 1).

No	Uncommon rules (heart disease)
1	{’atangina’, ’hcol’, ’peaklow’, ’maged’, ’F’, ’usloping’, ’hrnoraml’, ’exangina0’, **’fbsugar1’**}
2	{’atangina’, ’hcol’, ’maged’, ’F’, ’usloping’, ’hrnoraml’, ’exangina0’, **’fbsugar1’**}
3	{’atangina’, ’hcol’, ’peaklow’, ’maged’, ’F’, ’usloping’, ’exangina0’, **’fbsugar1’**}
4	{ ’atangina’, ’hcol’, ’peaklow’, ’maged’, ’F’, ’hrnoraml’, ’exangina0’, **’fbsugar1’**}
5	{’hcol’, ’peaklow’, ’maged’, ’F’, ’usloping’, ’hrnoraml’, ’exangina0’, **’fbsugar1’**}
6	{’atangina’, ’hcol’, ’peaklow’, ’F’, ’usloping’, ’hrnoraml’, ’exangina0’, **’fbsugar1’**}
7	{’atangina’, ’hcol’, ’F’, ’usloping’, ’hrnoraml’, ’exangina0’, **’fbsugar1’**}
8	{’atangina’, ’hcol’, ’peaklow’, ’F’, ’usloping’, ’exangina0’, **’fbsugar1’**}
9	{’hcol’, ’peaklow’, ’maged’, ’F’, ’hrnoraml’, ’exangina0’, **’fbsugar1’**}
10	{’atangina’, ’hcol’, ’maged’, ’F’, ’hrnoraml’, ’exangina0’, **’fbsugar1’**}

#### ST depression induced by exercise relative to rest (oldpeak)

The significance of the oldpeak value in the examination of noteworthy rules cannot be overstated, as it is present in 69 of the 163 notable rules under investigation. The transformation from a state of good health to one of rarity with heart disease is strongly indicative of heart disease, as the transition occurs when oldpeak is high and combined with these 69 common rules.

Our model places a high emphasis on the importance of a high oldpeak value, as it is associated with nearly 40% of the interesting rules. This suggests a strong connection between a high oldpeak value and an increased risk of heart disease. This information is valuable in identifying healthy rules that include these factors and serves as a significant alarm for potential heart disease in patients with high oldpeak values.

The presence of a high oldpeak value (indicated as ’peakhigh’) is a critical factor that triggers the transition from frequent, benign patterns to rare, high-risk patterns. This shift highlights the importance of monitoring oldpeak values closely, as they can serve as early warning signs for the onset of cardiovascular disease. The transformation from frequent to rare rules not only underscores the predictive power of oldpeak but also demonstrates the utility of our model in identifying these crucial changes in health status.

In Table 5, we present the top 10 rules that demonstrate the transformation of frequent rules (common in healthy patients) into rare rules as a result of high oldpeak values, signifying the development of heart disease. These rules illustrate the significant impact of oldpeak on cardiovascular risk and the value of our model in uncovering these patterns.

To illustrate how unexpected rare rules are generated, consider Rule 1 in Table 5. This rule indicates that if the cholesterol level is within the normal range, the patient’s age falls within the middle range, there is a high heart rate, fasting blood sugar levels are normal, the ST depression on the Resting Electrocardiogram presents an upsloping pattern, the chest pain type is non-anginal pain, and exercise-induced angina is absent, then a diagnosis of heart disease is made when the “Oldpeak” value is high.

This example underscores the importance of analyzing various factors in conjunction with oldpeak to make early and accurate diagnoses. Effective treatments aimed at reducing mortality associated with cardiovascular diseases can be developed by understanding these contributing factors. The availability of such frequent rules, particularly for individuals at high risk of developing heart disease when “Oldpeak” (ST depression induced by exercise relative to rest) is high, is of utmost importance. Hence, high oldpeak values often indicate ischemia or reduced blood flow to the heart muscle, which is a critical factor in the development of cardiovascular disease. By identifying patterns where high oldpeak values correlate with other risk factors, healthcare providers can develop more targeted interventions to manage and mitigate these risks.

The insights provided by our model highlight the need for comprehensive evaluations that consider the interplay of multiple factors. By identifying these critical patterns, healthcare professionals can better assess and manage patients at risk, ultimately improving outcomes and reducing the burden of cardiovascular diseases.

Overall, the presence of a high oldpeak value as a significant marker in our model underscores the importance of detailed cardiovascular assessments and proactive management strategies. The rules identified by our model provide a roadmap for clinicians to follow, ensuring that at-risk patients receive the necessary care to prevent the progression of heart disease. This proactive approach can lead to earlier interventions, better patient outcomes, and a reduction in the overall incidence of cardiovascular events.

Please note that this explanation is applicable to the remaining rules found in Table 5, emphasizing the broad applicability and significance of our findings across different patient profiles.

#### The slope of the peak exercise ST segment (ST slope)

The experimental results of the proposed model demonstrate that the ST Slope plays a significant role in the onset of cardiovascular disease, particularly when its value is (’flat’). According to the results, 28 out of 163 significant rare rules indicate cardiovascular disease when their ST Slope is ’flat’, compared to healthy patients (frequent rules without cardiovascular disease). This transition from frequent to rare rules signifies an increased likelihood of cardiovascular disease, indicating a critical shift in health status.

The flat ST Slope is particularly noteworthy because it reflects a significant modification in the underlying factors or conditions. A flat ST Slope during peak exercise typically indicates an abnormal response to physical stress, which can be a precursor to more serious cardiovascular issues. The occurrence of rarity along with specific rule attributes, such as a flat ST Slope, may act as a strong marker of cardiovascular risk. This requires further investigation of the causes and consequences of this transformation for health outcomes.

The top 10 interesting rules, displayed in Table 6, illustrate how this factor determines the rules that lead to cardiovascular disease. These rules deviate from the norms as their support falls and are often missed during frequent pattern mining. For example, a rule might indicate that a patient with normal cholesterol and no other significant symptoms, when combined with a flat ST Slope, suddenly falls into a high-risk category for heart disease.

Our proposed model excels in identifying these critical deviations, uncovering hidden patterns that are not apparent through traditional analysis. The identification of a flat ST Slope as a significant risk factor for cardiovascular disease highlights the importance of this symptom in clinical assessments. By recognizing the importance of a flat ST Slope, healthcare professionals can better assess the risk of cardiovascular disease in patients who might otherwise appear healthy.

As a result, the presence of a flat ST Slope is a vital factor in our model for detecting rare but significant rules that indicate cardiovascular disease. This insight underscores the importance of considering ST Slope in comprehensive cardiovascular risk assessments. The exceptional rules identified by our model, as shown in Table 6, provide a deeper understanding of the factors contributing to cardiovascular risk. These findings emphasize the necessity of thorough evaluations that include the ST Slope, enabling early detection and improved management of heart disease.

#### Type of chest pain: asymptomatic

A significant factor identified by our proposed model is the type of chest pain experienced by patients. Our experimental results have demonstrated that the occurrence of chest pain plays a crucial role in the development of heart disease. Specifically, among the 163 significant rare rules in otherwise healthy patients, 23 have been found to be at risk of heart disease when the chest pain is (’asymptomatic (asym)’). This indicates that the presence of asymptomatic chest pain, when combined with other common health indicators, significantly alters the patient’s health status, suggesting a strong likelihood of heart disease.

The presence of asymptomatic chest pain is particularly concerning because it often goes unnoticed by patients, delaying diagnosis and treatment. Our findings underscore the importance of identifying these subtle yet critical symptoms. The transition from frequent to rare rules signifies a substantial shift in health status, where the addition of asymptomatic chest pain to otherwise benign conditions results in an increased risk of heart disease.

The top 10 interesting rules, shown in Table 7, illustrate how this factor deviates from the norms and leads to rules that express patients with heart disease, despite their rarity. These rules highlight the critical nature of asymptomatic chest pain as a determinant in the onset of cardiovascular disease. For instance, a rule might indicate that a middle-aged individual with normal cholesterol and no other significant symptoms, when combined with asymptomatic chest pain, suddenly falls into a high-risk category for heart disease.

Our proposed model is effective in discovering these important rules that contribute to the development of heart disease. By focusing on the presence of asymptomatic chest pain, our model uncovers hidden patterns that are not evident in traditional analysis. This insight is invaluable for early detection and intervention, as it identifies patients who might otherwise be overlooked due to the absence of more obvious symptoms.

Therefore, the identification of asymptomatic chest pain as a significant risk factor for heart disease is a major finding of our study. The exceptional rules identified by our model, as shown in Table 7, provide a deeper understanding of the factors contributing to cardiovascular risk. These rules emphasize the importance of thorough clinical assessments that include the evaluation of subtle symptoms like asymptomatic chest pain. By incorporating these insights, healthcare professionals can improve early diagnosis and treatment, ultimately reducing the incidence and severity of heart disease.

#### Max heart rate

The importance of maximum heart rate in identifying unusual rules that diverge from typical frequent rules, which serve as standard beliefs, cannot be overstated. Our findings reveal that when the maximum heart rate is low, 12 out of 163 significant rare rules have been linked to cardiovascular disease in otherwise healthy individuals. These deviations from the norm occur specifically when their maximum heart rate (’hrlow’) is low. Consequently, these 12 unusual rules, represent deviations from the expected and are indicative of cardiovascular disease in patients.

Our proposed model has successfully uncovered these unique rules, highlighting critical contributors to the onset of cardiovascular disease despite their rarity. It is essential to note that all these distinctive rules apply to women. Furthermore, six of these distinctive rules are relevant to elderly women, specifically rules 1, 2, 8, 9, 11, and 12 as shown in Table 8. This suggests that elderly women with a low maximum heart rate are at a particularly heightened risk of developing cardiovascular disease, emphasizing the need for targeted interventions and monitoring in this demographic.

In contrast, when the maximum heart rate is high, our model does not identify any distinct rules directly linking this symptom to cardiovascular disease. However, it is crucial to emphasize that high maximum heart rate is associated with 59 interesting rare rules. These rules, although not directly caused by a high maximum heart rate, are linked to other significant factors that contribute to the development of cardiovascular disease. These factors include a high oldpeak, asymptomatic chest pain, and various other symptoms. This indicates that while a high maximum heart rate alone may not be a direct indicator, its presence alongside other risk factors can significantly increase the likelihood of cardiovascular disease.

This dual insight-highlighting the critical role of both low and high maximum heart rates in different contexts-demonstrates the robustness of our proposed model. It underscores the importance of considering maximum heart rate in comprehensive cardiovascular risk assessments. By identifying these rare but critical rules, our model provides valuable information that can aid in early diagnosis and targeted intervention, ultimately contributing to better patient outcomes and more personalized healthcare strategies.

In summary, the presence of a low maximum heart rate is a vital factor in our model for detecting rare but significant rules that indicate cardiovascular disease, particularly in women and elderly women. Conversely, a high maximum heart rate, while not directly causal, is associated with other risk factors that collectively indicate an increased risk of heart disease. These insights from our model emphasize the importance of a holistic approach in cardiovascular risk assessment, taking into account various interrelated factors to improve the accuracy and effectiveness of disease prediction and management.

#### Exercise-induced angina

Our research highlights the critical role of exercise-induced angina in identifying exceptional and atypical rules that deviate from established norms. Exercise-induced angina, indicated by a value of 1, is a condition where chest pain occurs during physical activity due to reduced blood flow to the heart. This factor has proven to be significant in our study.

Our findings indicate that when (’exercise-induced angina (’exangina1’) is present, 13 out of 163 rare rules exhibit a strong association with cardiovascular disease in otherwise healthy individuals. These 13 unusual rules, detailed in Table 9, contrast sharply with conventional norms and are effective in identifying patients with cardiovascular disease. These rules indicate that the presence of exercise-induced angina, combined with other factors, significantly alters the patient’s health status, leading to a higher risk of cardiovascular disease.

The detailed analysis of these rules reveals that the presence of exercise-induced angina, when combined with certain other health indicators, serves as a critical marker for cardiovascular disease. This demonstrates the power of our innovative model in uncovering important health insights that might be missed by conventional analysis. Despite their rarity, these rules provide valuable information for early diagnosis and prevention of heart disease.

On the other hand, our study found that when exercise-induced angina is absent (denoted by a value of 0), no exceptional rare rules are generated. This absence indicates that the lack of exercise-induced angina does not contribute to significant deviations from the norm, thus not highlighting any unusual patterns or risk factors for cardiovascular disease.

Thus, the presence of exercise-induced angina is a vital factor in our model for detecting rare but critical rules that point to cardiovascular disease. This insight underscores the importance of considering exercise-induced angina in clinical assessments and highlights its role in the early detection and management of heart disease. The exceptional rules identified by our model, as shown in Table 9, provide a deeper understanding of the factors contributing to cardiovascular risk.

#### Presence of fasting blood sugar

The experimental results have revealed the crucial function of (’fasting blood sugar (’fbsugar1’) in identifying exceptional and unconventional rules that diverge from established norms, especially those associated with frequently occurring rules without heart disease. Notably, all of these rare rules apply to women, as shown in the top 10 interesting rules illustrated in Table 10. Our findings demonstrate that when fasting blood sugar is present (indicated by a value of 1), 23 out of 163 rare rules exhibit a considerable association with cardiovascular disease in otherwise healthy women.

The presence of fasting blood sugar at elevated levels often coincides with other risk factors, such as high cholesterol and angina, particularly in women. This correlation underscores the heightened risk of developing heart disease when these factors are present. For example, women with high cholesterol and positive fasting blood sugar tests are at a significantly increased risk, especially if they also experience symptoms like angina or exercise-induced angina. This highlights the multi-faceted nature of cardiovascular risk, where the interaction between multiple factors compounds the overall risk.

Our analysis shows that these rare rules deviate significantly from conventional norms, effectively identifying female patients at risk for cardiovascular disease. This deviation from frequent patterns signifies a substantial change in the health status, indicating a critical shift towards disease when fasting blood sugar levels are high. The presence of high fasting blood sugar, as highlighted by our novel model, emerges as a crucial determinant in uncovering pivotal rules that contribute to the onset of cardiovascular disease in women. Despite their rarity and deviation from conventional norms, these rules provide essential insights for early diagnosis and intervention.

Conversely, the absence of a positive fasting blood sugar test (denoted by a value of 0) does not generate any exceptional rare rules. This suggests that normal fasting blood sugar levels do not significantly contribute to deviations from the norm, thereby not highlighting any unusual patterns or risk factors for cardiovascular disease. The absence of this factor indicates a lower risk profile, aligning with conventional medical understanding.

To summarize, the presence of fasting blood sugar is a vital factor in our model for detecting rare but critical rules that point to cardiovascular disease in women. This insight underscores the importance of considering fasting blood sugar levels in clinical assessments and highlights their role in the early detection and management of heart disease. The exceptional rules identified by our model provide a deeper understanding of the factors contributing to cardiovascular risk, particularly in female patients.

### Comparison with state-of-the-art methods

Our model, EPFHD-RARMING, is compared with two state-of-the-art methods used for heart disease prediction^8,43^. These methods represent the current advanced techniques in the field. The focus of our comparison is on identifying the factors contributing to heart disease, the interestingness and explainability of the results, and the comprehensiveness of the insights provided. This comparison aims to highlight the strengths and unique contributions of our approach in these specific areas.

#### Comparison with machine learning methods

In order to assess and evaluate our model, we compared it with a recent paper^8^ that used Catboost and other machine learning methods in order to detect and diagnose cardiovascular disease. Although the Catboost model achieved high accuracy, 91%, and significant F1-scores for heart disease prediction, it operates as a “black box”, making it difficult for clinicians to understand the underlying reasons for predictions. This lack of transparency can hinder trust and practical adoption in medical settings, where understanding the rationale behind predictions is crucial for effective treatment planning. In contrast, our proposed model, EPFHD-RARMING, generates clear, interpretable association rules. These rules provide medical professionals with comprehensible insights into the factors leading to heart disease, enabling them to make informed decisions and communicate effectively with patients. This interpretability is not just beneficial but essential for clinical decision-making, fostering greater confidence in the predictive model’s outputs.

While traditional ML models focus on optimizing prediction accuracy using large datasets, they often overlook rare but significant patterns. These models detect patterns present in the training data, potentially missing subtle and infrequent combinations of symptoms that could indicate future heart disease. This oversight can result in missed opportunities for early intervention. Our EPFHD-RARMING model excels at identifying rare rules with low support that deviate from frequent patterns, offering valuable insights into potential future risks. By capturing these rare but meaningful patterns, our model provides a proactive approach to disease management, highlighting vulnerable patterns that might develop into heart disease. By examining both frequent and rare association rules, we are able to gain a more comprehensive understanding of the factors associated with heart disease, thereby supporting early intervention and tailored patient care. This comprehensive approach addresses the critical need for interpretable and thorough predictive models in healthcare, ultimately leading to better patient outcomes, more informed clinical decisions, and the ability to anticipate and mitigate future health risks.

Our results also compare with another recent work^43^, which used a classification and regression tree (CART) algorithm for heart disease prediction, focusing primarily on model accuracy. While their approach identifies key risk factors through supervised learning, our novel method leverages rare rules to analyze unsupervised datasets, emphasizing interpretability and explainability. Unlike the supervised approach, our model uncovers detailed patterns and provides a comprehensive view of factors leading to heart disease, making the findings more actionable for healthcare professionals. Additionally, our model identifies patterns that may indicate future heart disease development, aiding in early detection and intervention. Our comprehensive and unsupervised approach makes our method highly adaptable to various domains and offers a more comprehensive understanding of cardiovascular health.

#### Identification and validation of key factors leading to heart disease

**Figure 9:**
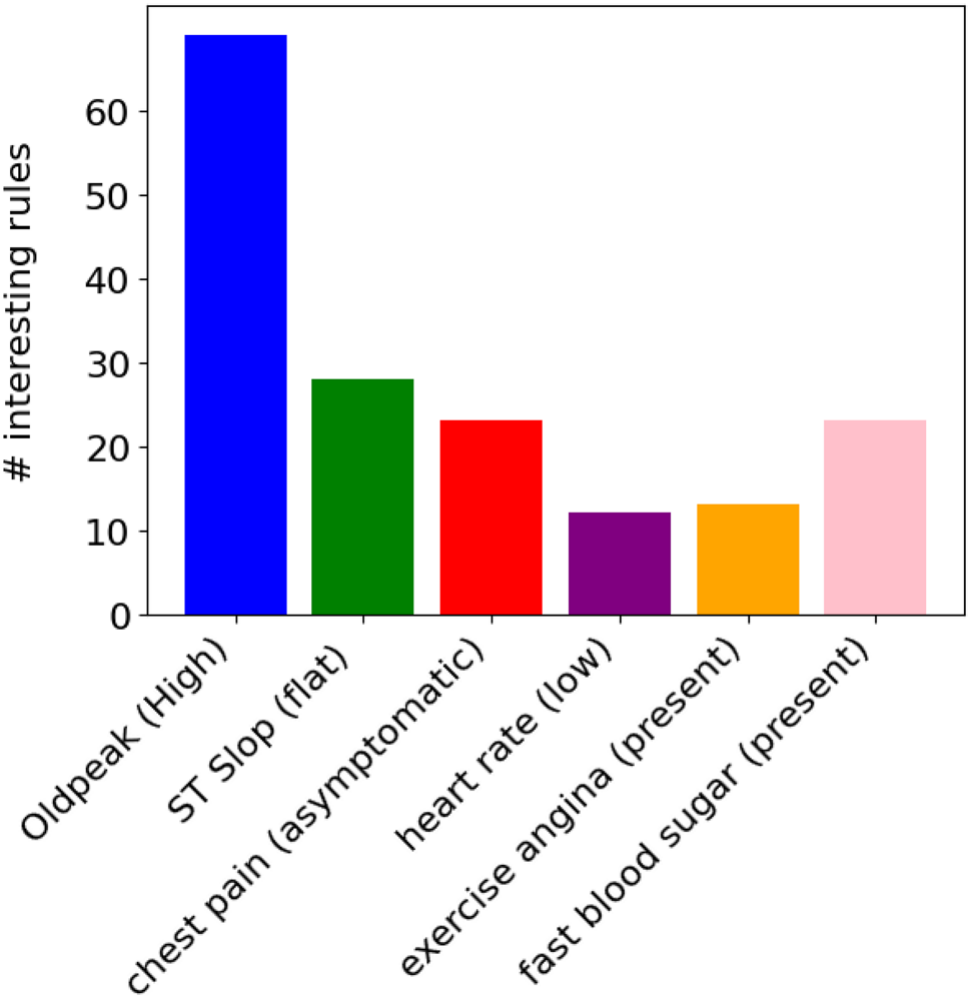
Factors contributing to the generation of interesting rules with heart disease as an outcome.

In our model, EPFHD-RARMING, we aim to uncover and highlight rare rules that contradict expectations, thereby leading to more remarkable discoveries. Our novel method is successful because it can extract interesting rules from hundreds of thousands of rules. Within this model, we employ well-established frequent rules as our grounding truth, representing widely accepted beliefs due to their high frequency of co-occurrence. Surprisingly, our model identifies specific factors that account for the transformation of common rules into rare ones, even with low support. These findings are particularly noteworthy, as demonstrated in our experiments. Several significant factors play a crucial role in the development of heart disease, including **ST depression induced by exercise relative to rest (Oldpeak), the slope of the peak exercise ST segment (ST Slope), asymptomatic chest pain, low heart rate, the presence of exercise-induced angina, and fasting blood sugar**. Figure 9 illustrates the factors that generate unexpected rules leading to heart disease.

The EPFHD-RARMING model not only unveils these previously unknown associations but also illuminates the intricate interplay of these factors, providing valuable insights into the development of heart disease in otherwise healthy individuals. The factors identified by our model have been further validated by applying multiple feature selection algorithms, which consistently identify the same variables as critical contributors. The convergence of methodologies across multiple approaches demonstrates the reliability and robustness of the factors identified by our model. According to our model, the factors contributing to heart disease include **’oldpeak’, ’ST slope’, ’chest pain type’, ’max heart rate’, ’exercise angina’, and ’fasting blood sugar’**. These results align with the most prominent feature selection methods, as illustrated in Fig. 3. This figure highlights the key features that contribute to the prediction of heart disease, as identified by multiple feature selection methods.

Additionally, a recent study^43^ has confirmed the significance of these factors, further attesting to their substantial impact on predictive modeling. This external corroboration serves as a strong endorsement of the accuracy and relevance of our proposed solution. Notably, our novel model uses rare association rule mining for this purpose, which serves as a robust option for future feature selection. By identifying these rare but significant patterns, our model provides a comprehensive tool for understanding and predicting heart disease, thereby facilitating early intervention and improved patient outcomes.

## Discussion

Our model, EPFHD-RARMING, utilizes an unsupervised method, specifically Association Rule Mining (ARM), which enhances the credibility of our findings. The unsupervised nature of our approach underscores the independence and objectivity of the model, allowing it to uncover patterns without the aid of predefined labels. This aspect of our model makes it highly adaptable and applicable to various domains beyond health, such as finance, marketing, and any field where identifying rare patterns is crucial. The alignment of our model’s findings with established feature selection algorithms, together with supporting evidence from recent studies, lends substantial evidence to the accuracy and correctness of our proposed solution. The utilization of an unsupervised method further emphasizes the model’s ability to autonomously identify and validate crucial factors in the absence of labeled data.

In contrast to conventional approaches in identifying factors that play a major role in prediction, our proposed model has effectively identified a diverse set of notable rules that can be summarized as follows:

- **Frequent rules relating to heart disease:** These rules closely reflect those derived from traditional methodologies, representing well-established rules associated with patients affected by heart disease.
- **Frequent rules facilitating early detection of heart disease:** Among the vast number of rules, our proposed model, EPFHD-RARMING, identified 163 interesting frequent rules that represent healthiness. The identification of these frequent rules that deviate to rare and interesting patterns upon the occurrence of one of the critical factors (such as ’oldpeak’, ’ST slope’, ’chest pain type’, ’max heart rate’, ’exercise angina’, and ’fasting blood sugar’) helps medical experts detect patients who may be at risk of developing heart disease. These vulnerable frequent patterns that deviate aid in the early determination of potential heart disease development.
- **Identifying risk factors:** Our model has been successful in identifying risk factors that contribute to the onset of heart disease.

The results of our model should be further investigated by domain experts and tested on more datasets to fully ascertain its effectiveness and importance. The validation of this model will assist in determining its generalizability and potential for wider application. By doing so, we can ensure that the insights provided by our model are robust and reliable, paving the way for its application in real-world scenarios.

Consequently, our groundbreaking model, EPFHD-RARMING, has demonstrated an exceptional ability to identify and prioritize rare rules that have low support, diverge from common rules, share similar antecedents, and contrast their outcomes with prevalent rules. The filtered rules provide valuable insights into the dataset’s patterns and deviations from the norm, enabling us to better comprehend exceptional cases and unforeseen associations. As a result, our paper presents a revolutionary model that transforms the discovery of heart disease. Conventional techniques often overlook critical risk indicators and fail to capture the intricate relationships between different factors. In contrast, our model, which utilizes an unsupervised tool, ARM, thoroughly analyzes the data, providing new insights into the complex cardiovascular health dynamics and yielding patterns that signify both health and potential risk. This innovative approach represents a significant breakthrough in the field, offering a more complete understanding of the multifaceted mechanisms underlying heart disease.

## Conclusion and future work

This article introduces EPFHD-RARMING, an unsupervised method developed to pinpoint crucial factors leading to heart disease. In contrast to conventional supervised techniques, our strategy employs rare association rule mining to improve efficiency and specificity in identifying predictive indicators. By defining frequent rules as fundamental beliefs, we were able to isolate rare yet significant rules that shed light on the onset of heart disease. Moreover, our method identifies sensitive frequent rules, which correspond to symptoms present in healthy individuals who may develop heart disease if the factors identified by our model are triggered. This predictive capability allows for early interventions, ultimately enhancing patient care.

EPFHD-RARMING effectively overcomes the limitations of conventional association rule mining, which often generates an excessive number of rules. Our approach narrows down this vast number to a more manageable 163 rules, focusing on rare, divergent factors with low support, and highlighting unique patterns and deviations within the dataset. This method confirms the value of our model in detecting key contributors to heart disease and enhances our understanding of exceptional and unforeseen cases within medical data.

The EPFHD-RARMING model’s future potential transcends its current boundaries. To further validate its versatility and effectiveness, our objective is to implement it across a wide array of datasets. Additionally, we plan to adapt the model for big data to boost its performance and scalability. By examining irregular patterns and pinpointing their underlying causes in different populations, we anticipate gaining more comprehensive insights into the factors contributing to heart disease.

The EPFHD-RARMING model’s potential extends beyond healthcare, with applications in other sectors, such as climate change. By identifying factors and trends that precipitate severe events, it can provide crucial insights for devising effective mitigation strategies. Ultimately, our aim is to broaden the model’s applicability not only for predicting heart disease but also for offering actionable insights for prevention, treatment, and crisis management across various fields.

While EPFHD-RARMING demonstrates potential, it is crucial to address several limitations. The present study’s dataset is limited, which restricts the generalizability of our findings. Consequently, future research involving larger and more diverse datasets is needed to validate and refine the model. Furthermore, the current method can be computationally intensive, especially with larger datasets, necessitating algorithm optimization to enhance performance and scalability. Although our model is interpretative, integrating it into clinical workflows seamlessly requires further testing and validation in real-world healthcare settings. External validation with additional datasets and clinical trials is also necessary to ensure the model’s robustness and reliability. In the future, improvements could focus on enhancing the model’s computational efficiency, expanding its applicability to other medical conditions, and developing strategies for seamless integration into clinical practice. These efforts will help establish the model’s utility and effectiveness across diverse healthcare scenarios.

In summary, the EPFHD-RARMING model represents a significant breakthrough in predictive modeling, providing a comprehensive, actionable understanding of heart disease. By harnessing rare association rule mining, our methodology overcomes the limitations of conventional methods, offering a robust tool for early intervention and improved patient outcomes. Future investigations should continue to explore and expand upon these techniques, unlocking their full potential across diverse applications and contributing to substantial advancements in various domains.

## Data Availability

The datasets^39^ that support the findings of this study are publicly available through IEEE. The heart disease research dataset is a comprehensive collection compiled from five independent sources: Cleveland, Hungarian, Switzerland, Long Beach VA, and Statlog (Heart) Data. This integration has resulted in one of the most extensive heart disease datasets available.
